# The future of fish in Africa: Employment and investment opportunities

**DOI:** 10.1371/journal.pone.0261615

**Published:** 2021-12-22

**Authors:** Chin Yee Chan, Nhuong Tran, Kai Ching Cheong, Timothy B. Sulser, Philippa J. Cohen, Keith Wiebe, Ahmed Mohamed Nasr-Allah

**Affiliations:** 1 WorldFish, Penang, Malaysia; 2 International Food Policy Research Institute, Washington, DC, United States of America; 3 WorldFish, Sharkia, Egypt; International Maize and Wheat Improvement Centre: Centro Internacional de Mejoramiento de Maiz y Trigo, MEXICO

## Abstract

One of the most pressing challenges facing food systems in Africa is ensuring availability of a healthy and sustainable diet to 2.4 billion people by 2050. The continent has struggled with development challenges, particularly chronic food insecurity and pervasive poverty. In Africa’s food systems, fish and other aquatic foods play a multifaceted role in generating income, and providing a critical source of essential micronutrients. To date, there are no estimates of investment and potential returns for domestic fish production in Africa. To contribute to policy debates about the future of fish in Africa, we applied the International Model for Policy Analysis of Agriculture Commodities and Trade (IMPACT) to explore two Pan-African scenarios for fish sector growth: a business-as-usual (*BAU*) scenario and a high-growth scenario for capture fisheries and aquaculture with accompanying strong gross domestic product growth (*HIGH*). Post-model analysis was used to estimate employment and aquaculture investment requirements for the sector in Africa. Africa’s fish sector is estimated to support 20.7 million jobs in 2030, and 21.6 million by 2050 under the *BAU*. Approximately 2.6 people will be employed indirectly along fisheries and aquaculture value chains for every person directly employed in the fish production stage. Under the *HIGH* scenario, total employment in Africa’s fish food system will reach 58.0 million jobs, representing 2.4% of total projected population in Africa by 2050. Aquaculture production value is estimated to achieve US$ 3.3 billion and US$ 20.4 billion per year under the *BAU* and *HIGH* scenarios by 2050, respectively. Farm-gate investment costs for the three key inputs (fish feeds, farm labor, and fish seed) to achieve the aquaculture volumes projected by 2050 are estimated at US$ 1.8 billion per year under the *BAU* and US$ 11.6 billion per year under the *HIGH* scenario. Sustained investments are critical to sustain capture fisheries and support aquaculture growth for food system transformation towards healthier diets.

## Introduction

Ensuring that a healthy and sustainable diet is available to 2.4 billion Africans by 2050 is one of the most pressing challenges facing Africa’s food systems [1–4]. The continent has struggled with a series of interconnected development challenges, particularly in fighting chronic food insecurity and overcoming pervasive poverty–the two foundational Sustainable Development Goals [5]. In Africa’s food systems, fish and other aquatic foods play a multifaceted role as a way of life, generating income, and providing a critical source of essential micronutrients, particularly for women and infants [1, 6–9]. Nevertheless, the current and future values of fish and aquatic foods in Africa are often overlooked in development research, policy and investment. It is argued that this oversight means multiple pathways to address malnutrition and food insecurity are underexplored [10].

Fish consumed in Africa are predominantly provided by capture fisheries sourced from rivers, large inland lakes and coastal systems [11]. Whereas aquaculture is one of the fastest growing food production sectors globally [12, 13], Africa contributed only 2.7% to the global aquaculture share in 2019. Nevertheless, the African aquaculture sector is maintaining double digit average annual growth rates in the last two decades in response to increasing fish demand in the continent [12]. Despite this growth, capture fisheries and aquaculture do not supply sufficient fish and there is a significant gap between fish supplies and consumer demand in Africa [1, 8]. Further, the fish supply gap is projected to widen due to a dramatic increase in fish demand, driven by rapid population and income growth, diet transformation resulting from urbanization, and changing consumer preferences [1, 8, 14]. In addition to these growing demands, unmet nutritional needs persist and continue to increase, particularly for women of reproductive age, children under the age of five and in the first 1000 days of life [15]. Increasing fish supply, reducing waste and loss, supporting intra-regional and international fish trade, and ensuring equitable distribution and access to fish are important strategies to address some dietary deficiencies and the costly individual and societal consequences [7, 16, 17].

Africa hosts regions that are amongst the most susceptible to global climate change [18]. Climate change projections [19–22] indicate that most of northern and southern Africa will experience high water stress while eastern, central, and western Africa will be subject to increasingly heavy rains and flooding [23, 24]. Changes in precipitation and temperature patterns due to climate change will create further stress in inland lake, river and oceanic ecosystems with ramifications on fish supply and the broader wellbeing of actors in the food systems. Climate change is projected to reduce the potential fisheries catch in the Exclusive Economic Zones (EEZs) in Africa [21]. Coupling with climate change impacts, the activities of foreign fishing vessels in African EEZs are also likely to impact fish availability and access in Africa [25]. In sum, there are growing uncertainties and daunting challenges associated with the future of Africa’s food systems associated with large-scale drivers that operate outside and within fisheries and aquaculture systems. These challenges, amplified by the unprecedented COVID-19 pandemic have led the governments of African and regional organizations to determine the potential investment opportunities and interventions in fisheries and aquaculture to address food and nutrition security [26, 27].

Public and private sector investment will be critical to secure diverse supplies of fish and other aquatic foods from capture fisheries and aquaculture. Whilst contributing to food and nutrition security in Africa will require four simultaneous strategies (i.e., increasing fish supply, reducing waste and loss, supporting fish trade, and ensuring equitable distribution and access), in this paper we focus on fish supply that could be achieved by increasing production. To date, there are no estimates of investment and potential returns for domestic fish production in Africa. To contribute to policy debates about the future of fish in Africa, we develop two scenarios; *business-as-usual* (*BAU*) and *high capture fisheries and aquaculture with stronger GDP growth* (*HIGH*). We first project future fish supply and demand in Africa to 2050 using the International Model for Policy Analysis of Agriculture Commodities and Trade (IMPACT). Second, we conduct post-model analysis to extrapolate future potential direct (capture fisheries and aquaculture) and indirect employment that would be associated with the *BAU* and *HIGH* scenarios. Finally, we estimate future aquaculture production value and investment costs required to achieve *BAU* and *HIGH* scenarios.

## Materials and methods

**Fig 1:**
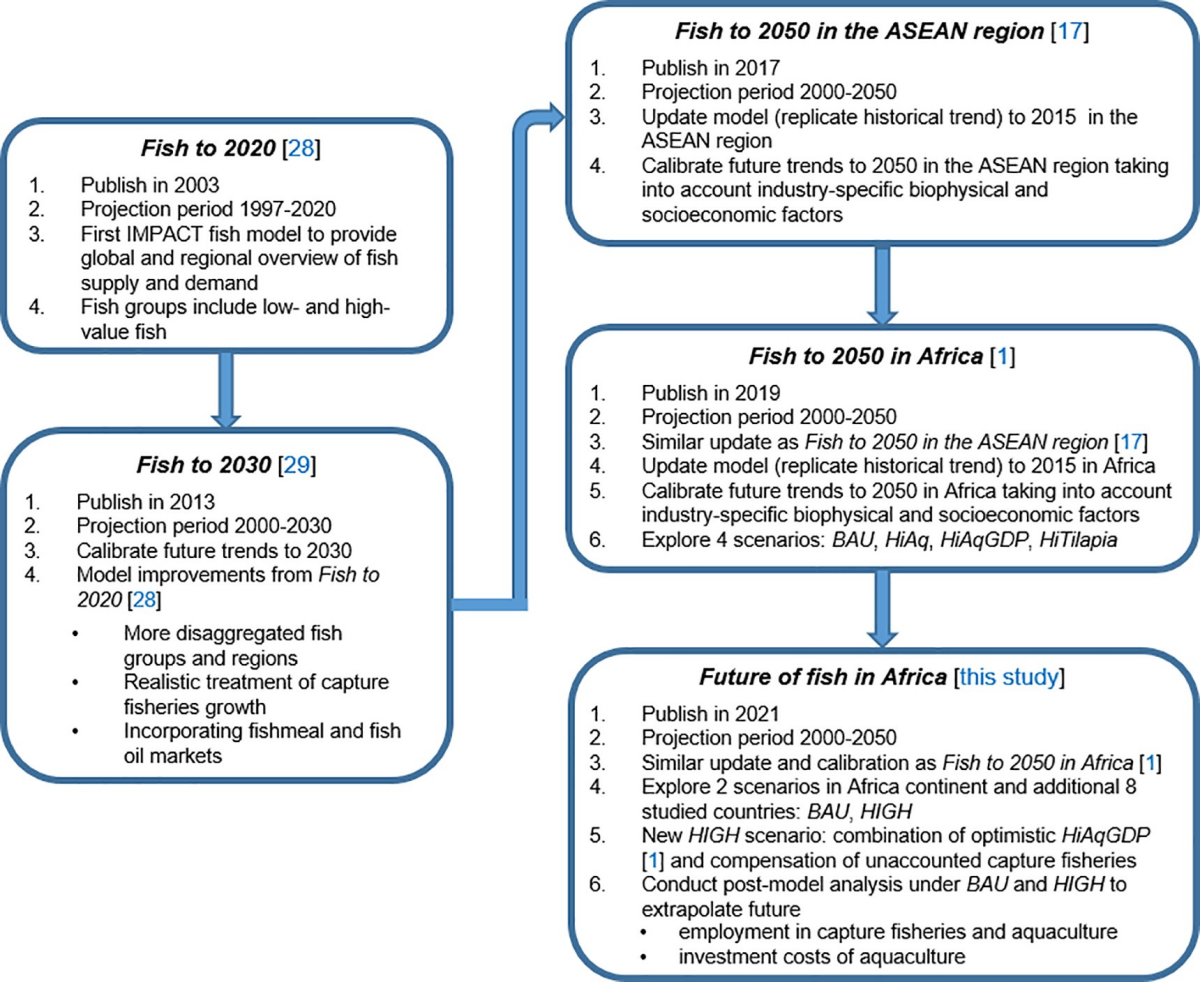
Chronological model improvement and analysis using IMPACT fish model.

To provide more comprehensive and consistent outlooks and prospects of fish and aquatic food systems, efforts have been made to integrate fish into foresight modeling of agriculture and livestock commodities. We apply the IMPACT fish model developed by International Food Policy Research Institute (IFPRI), which is a partial equilibrium economic model containing a system of equations for analyzing baseline and alternative scenarios for fish demand, supply, trade and prices at global, regional and country level in responding to future changes such as income, population and technological progress. [28, 29]. Previous application of the model by the World Bank in “*Fish to 2030”* report [29] used global historical data up through 2009 to develop business-as-usual (*BAU*) scenario. The projection from that model underestimated the 2010–2015 historical trend of capture fisheries and aquaculture production. To address these shortcomings, we re-calibrate the model with recent dataset and parameters of fish production, consumption, trade, population and GDP compiled from FAO, UN and IFPRI databases [4, 12, 30]. Specifically, we revisited the productivity growth assumptions of the model, using expert knowledge informed by fisheries and aquaculture specific biophysical and socio-economic factors and fish management and production targets defined by national governments [1]. The progressive improvement of IMPACT fish model used to project future Africa’s fish sector is illustrated in Fig 1.

In this study, we focused on eight African nations: Egypt, Ghana, Kenya, Malawi, Nigeria, Tanzania, Uganda and Zambia. We selected these countries because they are 1) nations projected to face the largest shortfalls in fish supply relative to demands, 2) experience high rates of fish consumption, and 3) are amongst the nations experiencing relatively rapid growth in aquaculture (Table 1). These eight countries are home to 40% of Africa’s total population but produce over 95% of aquaculture and 30% of capture fisheries production (by volume) in the continent in 2019. About half of fish consumed in Africa is by these eight countries, suggesting slightly higher per capita fish consumption rates than elsewhere in Africa [31]. Among these eight countries, Uganda, Tanzania, Malawi, Kenya, and Ghana are classified by Food and Agriculture Organization (FAO) as low-income food-deficit countries [32].

**Table 1:** Contribution of fish to food security in Africa and the world.

Indicator	Year	Egypt	Ghana	Kenya	Malawi	Nigeria	Tanzania	Uganda	Zambia	Studied countries	Africa	World
**Demographic and socio-economic status** ^**a**^												
Population (million)	***2020***	102.3	31.1	53.8	19.1	206.1	59.7	45.7	18.4	536.3	1,340.6	7,794.8
Population average annual growth (%)	***2010–2020***	2.1	2.3	2.5	2.8	2.7	3.0	3.5	3.1	2.6	2.6	1.1
Urban population (%)	***2020***	42.8	57.3	28.0	17.4	52.0	35.2	25.0	44.6	42.5	43.3	56.2
GDP per capita (current US$)	***2020***	3,548	2,329	1,838	625	2,097	1,077	817	1,051	2,047	1,789	10,926
GDP average annual growth (%)	***2010–2020***	5.2	8.4	9.5	5.6	1.8	6.9	3.5	-0.5	4.0	1.6	2.5
Undernourishment (%)	***2019***	5.4	6.1	24.8	17.3	14.6	25.1	n.a.	n.a.	14.6	17.7	8.9
Unemployment (% total labor force)	***2020***	10.5	4.5	3.0	6.0	9.0	2.2	2.4	12.2	7.1	7.7	6.5
	**Year**	**2017**	***2016***	***2015***	***2016***	***2018***	***2017***	***2016***	***2015***		*** ***	***2017***
Population below US$1.90 a day (%)		3.8	12.7	37.1	69.2	39.1	49.4	41.3	58.7	n.a.	n.a.	9.3
**Contribution of fish to food supply** ^**b**^												
Total fish production (thousand tonnes)	***2019***	2,039	445	144	163	1,115	487	706	136	5,235	12,385	177,834
Share of aquaculture production (%)	***2019***	80.5	11.8	12.9	5.1	26.0	3.4	14.6	28.3	41.4	18.4	48.0
Aquaculture average annual growth (%)	***1999–2019***	10.4	15.6	22.9	14.1	13.8	24.7	30.9	11.7	11.3	11.1	5.2
Capture fisheries average annual growth (%)	***1999–2019***	-0.3	-1.2	-2.4	6.3	3.0	2.1	5.0	1.9	1.6	2.3	0.04
**Contribution of fish to food and nutritional status** ^**c**^												
Fish consumption (kg/capita/year)	***2018***	23.2	24.8	3.0	11.9	8.9	6.8	10.9	11.7	12.1	10.3	20.2
Fish protein (g/capita/day)	***2018***	6.6	8.0	0.9	3.5	2.6	2.2	3.3	3.5	3.6	3.0	5.6
Animal protein (g/capita/day)	***2018***	26.4	15.4	14.9	9.3	7.3	12.1	12.3	13.7	13.5	15.2	32.9
Fish/animal protein (%)	***2018***	25.0	52.2	5.7	37.6	35.3	18.5	26.5	25.3	29.1	20.0	16.9
**Contribution of aquaculture value** ^b^												
Farm-gate value (million US$)	***2019***	2,862	190	64	38	833	62	242	105	4,395	4,857	259,548
Farm-gate price (US$/kg)	***2019***	1.7	3.6	3.5	4.6	2.9	3.8	2.3	2.7	2.0	2.1	3.0

Author’s computation from data source ^a^UN [4]

^a^World Bank [33]

^b^FishStatJ [12, 31]

and

^c^FAO [34].

We consulted 76 experts from Egypt (43%), Nigeria (32%), Tanzania (15%), Zambia (4%), Ghana (2%), Kenya (2%) and South Africa (2%), during five stakeholder consultation workshops organized in Egypt, Nigeria, and Tanzania from 2017 to 2019. We sought the input of experts from government (27%), non-governmental organizations (47%), academia (13%), and private sector (13%) from different fields of expertise, covering fisheries, aquaculture, nutrition, gender, trade and economics to update and refine the model, explore alternative scenarios, validate projection results, verify the employment and investment dataset, and verify the post-model employment and investment estimation. The consensus had reached when no further comments from the stakeholders during consultation process.

### Scenario analysis

We developed two scenarios in this study. The first scenario was business-as-usual future (*BAU*) which was characterized by a set of model parameters that reflect a continuation of past trends into the future. We had determined these trends from the regional experts we had gathered in the consultation workshops. In our *BAU* scenario, we use the Shared Socioeconomic Pathway (SSP) 2 [35], which assumes economic development continues but is not uniform, environmental degradation continues, but at a slowing pace compared to historical trends, and climate change presents moderate challenges to both adaptation and mitigation. Under the *BAU* scenario, African economies are assumed to have a low annual income growth rate of 2.9% from 2015–2050. This *BAU* scenario replicated projection results reported in our previous study [1].

The second alternative scenario is called *high capture fisheries and aquaculture with stronger GDP growth* (*HIGH*) assumes high aquaculture growth rates being driven by substantial investment in the industry. The model was calibrated such that aquaculture output grows at 12.7% over the 2015–2030 period (a relative improvement compared to the 10.6% aquaculture output growth observed from the 2005 to 2015 period). This is achieved by adjusting the model’s exogenous productivity growth rates of the top five aquaculture producing countries in Africa (Egypt, Nigeria, Uganda, Ghana, and Zambia) for key selected species farmed in Africa (59% tilapia, 11% catfish and 11% mullet) from 2015–2050. For capture fisheries, FAO statistics [12] reported that, in 2017, Africa produced 7.0 million tonnes of marine capture fisheries and 3.0 million tonnes of inland fisheries. However, Kolding *et al*. [36] estimated substantially higher production (about 20 million tonnes) from inland fisheries based on the total freshwater resources available in Africa (e.g., lakes, rivers, reservoirs, flood plains, and swamps). Given this disparity in capture fisheries estimates, we postulate in this scenario that the potentially unaccounted capture fisheries quantities are accrued to the existing *BAU* projections. To investigate the low per capita fish consumption in Africa, this scenario also assumes an increase in per capita incomes. Under the *HIGH* scenario, we set a moderate optimistic annual income growth rate of 4.8% per year compared to SSP 2 of 2.9% under *BAU*.

### Post-model estimation of employment

Employment is a key indicator for assessing socio-economic contributions of the fisheries and aquaculture sectors to food, incomes, and livelihoods. Yet, due to the informal and dispersed nature of much of the sector, quality employment data are limited for both capture fisheries and aquaculture and their value chains. To estimate direct and indirect employment in the *BAU* and *HIGH* scenarios, we reviewed national employment data from global data sets [37, 38] and national sources [39–54]. We adopted the definitions of direct and indirect employment used by the FAO [38] which suggest a full time employee is one that received 90% of their livelihood or spends 90% of their time in that occupation; a part time employee between 30–90%, an occasional employee less than 30%, and indirect jobs are “those associated with ancillary activities such as the building of infrastructure (ponds, cages, tanks, etc.), feed and seed production, manufacturing of fish processing equipment, packaging, marketing, and distribution”. We also take indirect employment equals direct employment (full-time equivalent number of jobs) times employment multiplier presented in Table 4.

To account for inconsistent data, we further adjusted direct and indirect employment data to better reflect our experts’ assessment of labor productivity and average employment multiplier during stakeholder consultation. We compiled the labor productivity data and used it to estimate future employment through capture fisheries. Future employment in the aquaculture sector was based on the increasing trend of labor productivity in Africa’s aquaculture sector observed over the past three years [37]. Assumptions for direct employment in aquaculture include a labor productivity/efficiency increase of 10% from 2018 to 2030, and again from 2030 to 2050 in Africa and the studied countries. This assumption aligns with the potential technology advancement to reduce labor requirements in aquaculture and fisheries production in the future.

### Post-model estimation of aquaculture investment costs

For investment cost extrapolation, due to data limitations in capture fisheries, we focused explicitly on aquaculture alone to determine the size of investment needed to meet the *BAU* and *HIGH* projections of production. Aquaculture production values for the base year 2016 (except Uganda and Zambia base year in 2014) were computed using commodity prices collected from literature for each country. Production values of the base and future projections in 2030, and 2050 under the *BAU* and *HIGH* scenarios were converted to 2010 constant US dollar using the World Bank’s consumer price index. The investment needed to support projected value was built on key variable input costs such as seed (i.e., the broodstock, hatchlings, or fry that are spawned or caught from the wild), feed (i.e., a combination of ingredients made into a single feed for growing fish), and labor (i.e., fish farmers with full time equivalent number of direct jobs). The magnitude of costs for each scenario was ascertained through literature review and validated through our expert consultation workshops. The costs of inputs were determined using farm-gate prices, average productivity, average market size, survival rate, stocking density, feed conversion ratio, average wages, input prices, and profit margins (Table 2). We present the variation of these inputs information in single value in Table 2 after validation via the stakeholder consultation process. Future input costs were converted to constant US$ in 2010 using the consumer price index (i.e., dollar values are divided by the consumer price index of that year, and then multiplied by the index of 2010). Fixed costs such as infrastructure investment costs, and public spending for aquaculture research, development and extension, were not included in our estimation due to data limitations.

**Table 2:** Key parameters used for estimating the quantity and cost of key inputs in studied countries.

Base year	Egypt	Ghana	Kenya	Malawi	Nigeria	Tanzania	Uganda	Zambia
	2016	2016	2016	2016	2016	2016	2014	2014
Farm-gate price (US$/kg)	0.95	1.45	2.12	0.98	1.41	1.96	1.99	1.78
Feed conversion ratio (FCR)	1.12	2	1.5	1.8	1.3	1.5	1.5	1.7
Productivity (tonne/ha)	10.8	2.9	5	1.8	4	10	10	1.1
Average market size (g/fish)	300	400	400	-	800	-	-	-
Survival rate (%)	90	80	80	-	80	-	70	90
Stocking density (1000 pieces/ha)	40	-	15.6	6	6.3	30	2.5	2.8
Seed price (US$/1000 pieces)	5.6	39	62.6	6.6	57	90	70	43
Feed price (US$/kg)	0.38	0.48	0.63	0.33	0.57	0.81	0.56	0.30
Average wage (US$/year)	713	145	188	33	509	175	211	339
Profit margin (%)	34	15	23	19	28	11	37	23
References	[51, 55]	[39, 56–64]	[44, 52, 65–73]	[40, 41, 54, 74–76]	[70, 77–81]	[45, 48, 49, 82–86]	[43, 53, 87]	[46, 50, 88–90]

All values are converted to constant US$ in 2010 based on World Bank’s consumer price index.

## Results

### Scenarios

Previous projections of *BAU* scenario [1] had suggested that African capture fisheries and aquaculture production will grow at 0.2% and 1.3%, respectively, from 2015 to 2050. Despite the higher growth rate of aquaculture, capture fisheries in Africa will continue to be the main contributor to total fish production until 2050, though Egypt is a notable exception. Driven by high population growth and low GDP growth, per capita fish consumption in Africa is projected to gradually drop from 10.0 kg/year in 2015 to 7.7 kg/year in 2050 under this scenario (Table 3).

**Table 3:** IMPACT fish model scenario projection of fish production and per capita fish consumption for Africa in 2015, 2030, and 2050.

Region	Scenarios	Capture fisheries (million tonnes)			Aquaculture (million tonnes)			Per capita fish consumption (kg/person/year)		
		2015	2030	2050	2015	2030	2050	2015	2030	2050
**Africa**	*BAU*	8.7	9.0	9.2	1.8	2.4	2.9	10.0	8.5	7.7
	*HIGH*		15.8	16.0		11.0	18.8		12.1	14.0

Under the *HIGH* scenario, the total capture fisheries production is projected to be 76% and 74% higher in 2030 and 2050 compared to the *BAU* scenario. This is driven by better accounting for inland capture fisheries production, rather than substantial increases in capture fisheries production. The total aquaculture production is projected to be 350% and 558% higher in 2030 and 2050, respectively, compared to the *BAU* projections (Table 3). With these high growth assumptions, the aquaculture production in Africa will likely surpass capture fisheries production by 2050. High GDP growth will enable purchasing power to increase per capita fish consumption from 10 kg in 2015 to 12 kg in 2030 and 14 kg in 2050 (Table 3).

### Employment in fish sectors

Table 4 depicts that, overall, African capture fisheries and aquaculture sectors are estimated to sustain 20.7 million jobs (direct and indirect employment) in 2030, and generate 21.6 million jobs by 2050 under the *BAU* scenario. Direct employment in Africa’s fish sector is estimated to remain relatively constant and only grow from 5.6 million in 2030 to 5.8 million in 2050 in the *BAU* scenario. In contrast, under the *HIGH* scenario, direct employment in capture fisheries and aquaculture will be more than double in comparison to the BAU, reaching 12.2 million by 2050, where for every person directly employed in the sector, 2.6 people will be indirectly employed. By 2050, capture fisheries and aquaculture sectors will sustain 58.0 million jobs. Under the *BAU* and *HIGH* scenarios, the total direct and indirect employment for the fish sector will represent 0.9% and 2.4%, respectively, of the total projected 2.4 billion African in 2050 [4].

**Table 4:** Estimated direct and indirect employment of Africa’s fish food system for *BAU* and *HIGH* scenarios in 2030 and 2050.

Country	Scenarios	Fish production (thousand tonnes)		Direct labor productivity (tonnes/worker)		Direct employment (thousand)		Average employment multiplier		Indirect employment (thousand)		Total direct and indirect employment (thousand)	
		2030	2050	2030	2050	2030	2050	2030	2050	2030	2050	2030	2050
**Africa**	*BAU*	11,439	12,064	2.0	2.1	5,630	5,774	2.6	2.7	15,035	15,855	20,665	21,629
	*HIGH*	26,784	34,816	2.4	2.8	11,049	12,230	3.2	3.7	35,202	45,758	46,251	57,988
**Egypt**	*BAU*	1,924	2,169	12.4	13.4	156	161	1.8	2.0	288	324	443	485
	*HIGH*	4,977	7,632	12.8	14.1	389	540	1.7	2.0	680	1,078	1,069	1,617
**Ghana**	*BAU*	369	389	1.4	1.4	271	276	2.1	2.2	565	597	835	872
	*HIGH*	1,147	1,722	1.7	2.2	660	790	2.7	3.3	1,757	2,639	2,417	3,429
**Kenya**	*BAU*	209	213	1.9	1.9	111	113	2.0	2.0	217	222	328	335
	*HIGH*	618	630	2.7	2.6	228	242	2.8	2.7	644	656	872	897
**Malawi**	*BAU*	133	136	1.0	1.0	135	137	4.0	4.0	534	545	669	682
	*HIGH*	397	404	1.0	1.0	400	406	4.0	4.0	1,595	1,622	1,995	2,028
**Nigeria**	*BAU*	1,441	1,638	1.3	1.3	1,137	1,266	0.9	0.9	1,005	1,142	2,142	2,409
	*HIGH*	2,072	2,396	1.2	1.3	1,749	1,903	0.8	0.9	1,445	1,670	3,194	3,573
**Tanzania**	*BAU*	341	341	1.8	1.8	192	192	1.3	1.3	247	247	439	439
	*HIGH*	1,087	1,088	1.8	1.8	602	604	1.3	1.3	786	787	1,409	1,416
**Uganda**	*BAU*	639	669	3.6	3.6	179	183	3.9	4.0	693	726	872	909
	*HIGH*	2,070	2,151	3.4	3.4	616	632	3.6	3.7	2,245	2,333	2,861	2,965
**Zambia**	*BAU*	115	124	1.3	1.3	92	96	0.5	0.5	49	52	141	148
	*HIGH*	418	474	1.2	1.3	347	377	0.5	0.5	177	200	524	578

For direct employment in African aquaculture, even with increasing average labor productivity from 5.8 tonnes/worker in 2030 to 6.3 tonnes/worker in 2050, fish farmers are projected to increase to 0.3 million under *BAU* and sharply increase to 1.1 million under *HIGH* by 2050 due to the 71% increase in aquaculture production. Among the eight studied countries, Egypt has the highest labor productivity of 13–15 tonnes/worker, followed by Uganda, Nigeria, Ghana, and Zambia. Conversely, Malawi, Tanzania, and Kenya have relatively lower labor efficiency with less than one tonne/worker (Table 5).

**Table 5:** Estimated direct employment of Africa’s aquaculture sector for *BAU* and *HIGH* scenarios in 2030 and 2050.

Country	Scenarios	Aquaculture production (thousand tonnes)		Labor productivity (tonnes/worker)	Labor productivity (tonnes/worker)	Direct employment	
		2030	2050	2030	2050	2030	2050
**Africa**	*BAU*	2,439	2,864	5.8	6.3	424,368	452,866
	*HIGH*	10,984	18,816			1,910,711	2,975,598
**Egypt**	*BAU*	1,594	1,843	13.3	14.6	120,303	126,449
	*HIGH*	4,550	7,210			343,318	494,569
**Ghana**	*BAU*	85	102	3.9	4.2	21,929	24,067
	*HIGH*	558	1,122			144,856	264,663
**Kenya**	*BAU*	33	38	0.6	0.6	60,080	62,722
	*HIGH*	33	45			60,063	73,717
**Malawi**	*BAU*	8	9	0.8	0.9	9,328	10,292
	*HIGH*	8	11			9,328	12,124
**Nigeria**	*BAU*	445	526	4.5	5.0	98,846	106,190
	*HIGH*	500	707			111,188	142,952
**Tanzania**	*BAU*	7	8	0.8	0.9	9,010	9,315
	*HIGH*	7	10			9,005	10,822
**Uganda**	*BAU*	134	154	5.4	5.9	25,071	26,207
	*HIGH*	136	184			25,468	31,305
**Zambia**	*BAU*	29	34	1.8	1.9	16,379	17,610
	*HIGH*	61	103			34,658	53,325

**Fig 2:**
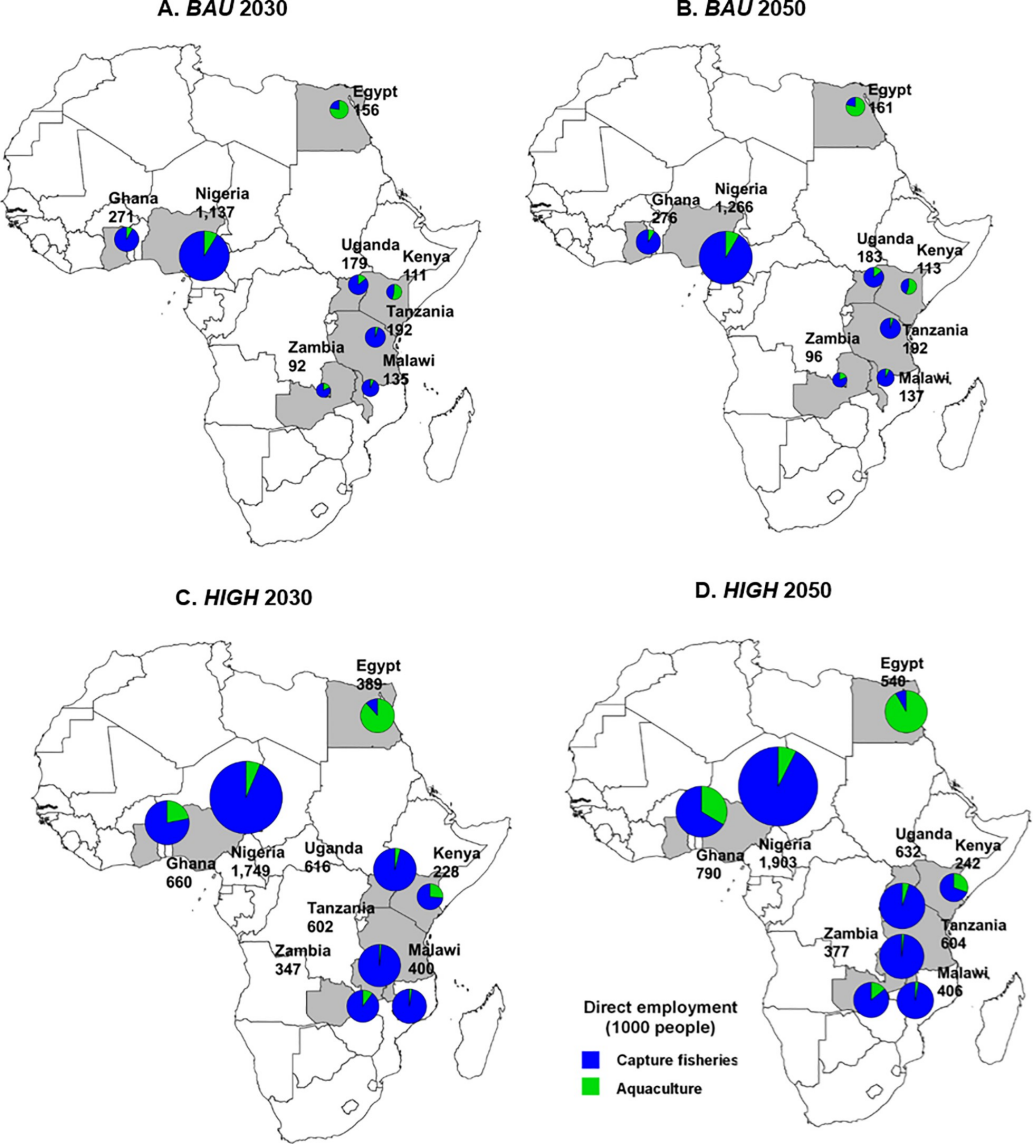
Direct employment of capture fisheries and aquaculture under *BAU* scenario in 2030 (A), *BAU* scenario in 2050 (B), *HIGH* scenario in 2030 (C), and *HIGH* scenario in 2050 (D).

**Fig 3:**
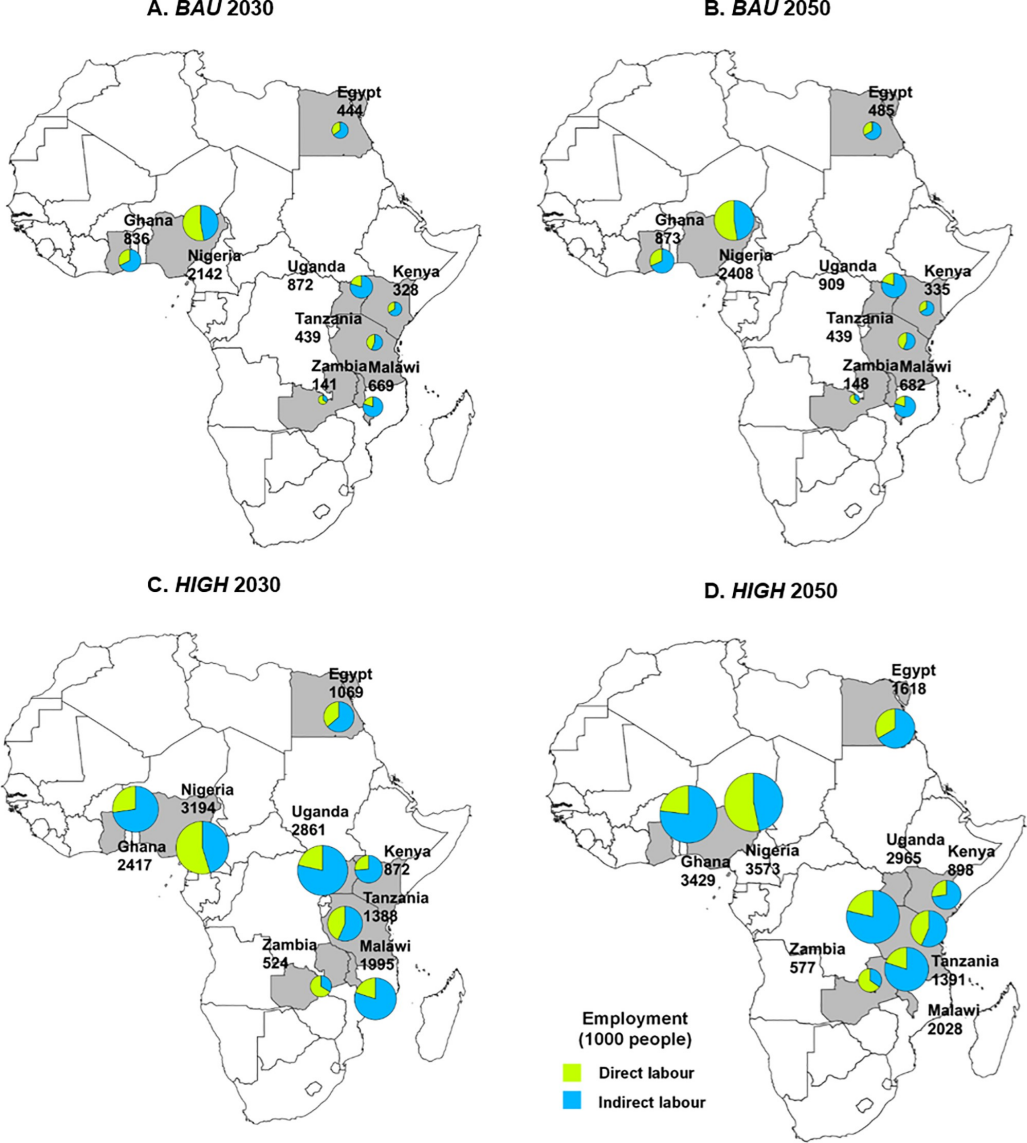
Direct and indirect employment of fish sector under *BAU* scenario in 2030 (A), *BAU* scenario in 2050 (B), *HIGH* scenario in 2030 (C), and *HIGH* scenario in 2050 (D).

Employment generated by capture fisheries contributes to over 90% of the total jobs in the African fish sector under *BAU*. This is mainly due to higher capture fisheries output but lower labor productivity as compared to aquaculture. Among the studied countries, Egypt again has the highest labor efficiency in capture fisheries of 9.3 tonnes/worker (Tables 5 and 6). Egypt is the only country that has a higher proportion of jobs generated by aquaculture than capture fisheries in the *HIGH* scenario (Fig 2). Nigeria is among the studied countries that generates the highest total employment in the fish sector, particularly in the capture fisheries sector. Only two countries—Nigeria and Zambia—have average employment multipliers less than one, resulting in the proportion of indirect employment being less than half of the total employment in the fish sector (Fig 3).

**Table 6:** Estimated direct employment of Africa’s capture fisheries sector for *BAU* and *HIGH* scenarios in 2030 and 2050.

Country	Scenarios	Capture fisheries production (thousand tonnes)		Labor productivity (tonnes/worker)	Direct employment	
		2030	2050		2030	2050
**Africa**	*BAU*	9,000	9,200	1.7	5,205,551	5,321,230
	*HIGH*	15,800	16,000		9,138,634	9,254,313
**Egypt**	*BAU*	330	326	9.3	35,328	34,869
	*HIGH*	427	422		45,690	45,231
**Ghana**	*BAU*	284	287	1.1	248,730	251,645
	*HIGH*	588	600		515,164	525,665
**Kenya**	*BAU*	176	175	3.5	50,456	50,415
	*HIGH*	585	585		168,211	168,170
**Malawi**	*BAU*	125	126	1.0	125,534	126,717
	*HIGH*	389	393		385,213	393,786
**Nigeria**	*BAU*	996	1,113	1.0	1,038,293	1,159,992
	*HIGH*	1,572	1,688		1,638,155	1,759,854
**Tanzania**	*BAU*	334	333	1.8	183,254	182,948
	*HIGH*	1,079	1,079		592,958	592,680
**Uganda**	*BAU*	505	515	3.3	154,162	157,281
	*HIGH*	1,934	1,967		590,683	600,731
**Zambia**	*BAU*	86	90	1.1	75,607	78,438
	*HIGH*	357	370		312,323	324,017

### Aquaculture production values and investment costs

**Fig 4:**
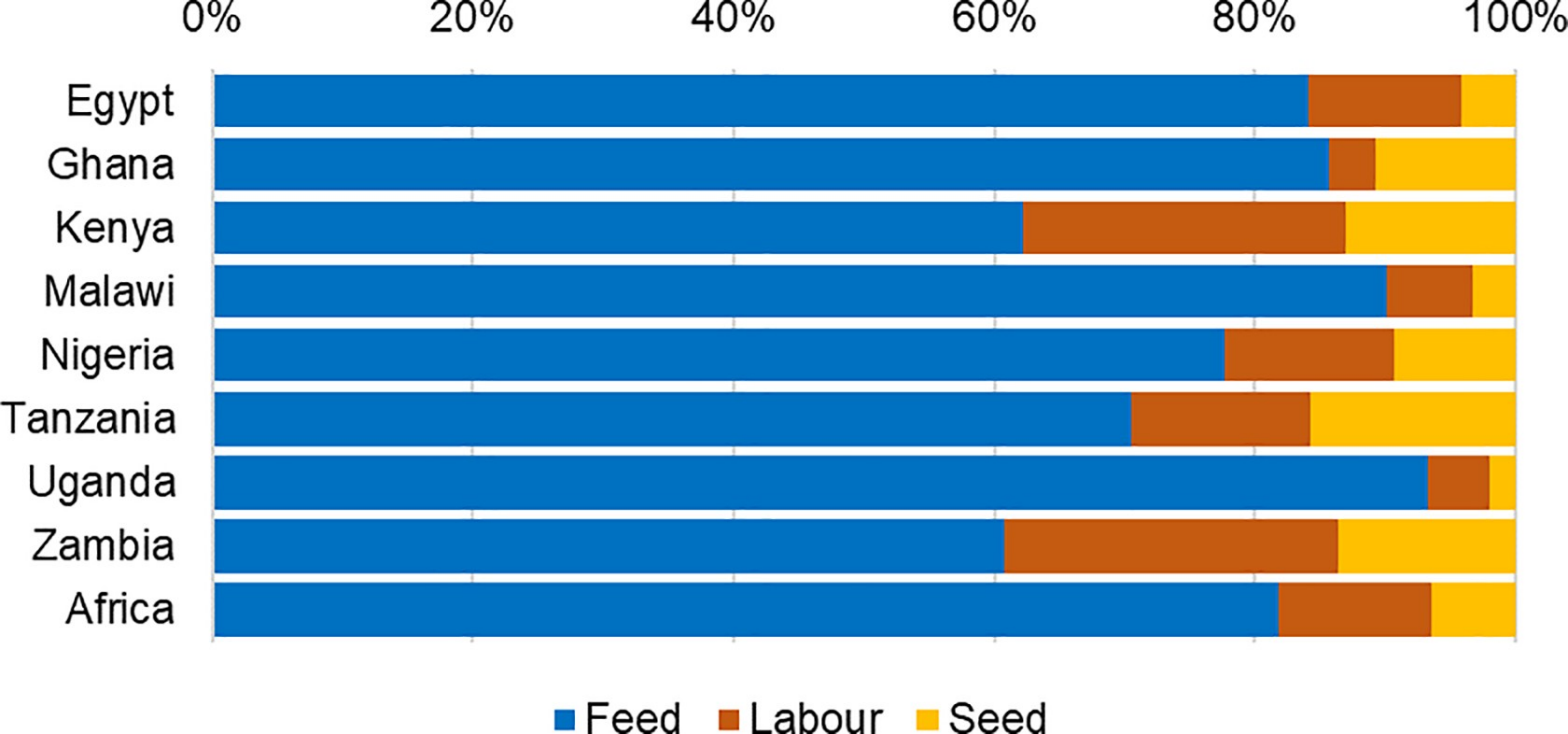
Cost structure of key input production costs of aquaculture in Africa and studied countries.

Under the *BAU* scenario, Africa’s aquaculture production is projected to reach 2.4 million tonnes, valued at US$ 2.8 billion, and 2.9 million tonnes, valued at US$ 3.3 billion in 2030 and 2050, respectively. Farm-gate investment costs for three key variable inputs of feed, labor, and fish seed to realize aquaculture production in 2030 and 2050 are shown in Table 7. The investment costs are projected to increase to US$ 1.6 billion and US$ 1.8 billion in 2030 and 2050, respectively, to achieve the projected aquaculture outputs in those years. Of the three key variable costs estimated, feed costs account for 81% to 84%, labor costs range from 10% to 12%, and fish seed costs a little over 6% (Fig 4). The investment cost structure is likely to remain the same, unless there are technological innovations in the fish feed and seed sectors, resulting in a substantial decrease in feed costs.

**Table 7:** Annual output value and key inputs costs of Africa’s aquaculture for *BAU* and *HIGH* scenarios in 2030 and 2050.

Country	Scenarios	Aquaculture production values (million US$)		2030 farm-gate costs (million US$)				2050 farm-gate costs (million US$)			
		2030	2050	Feed	Labor	Seed	Total	Feed	Labor	Seed	Total
**Africa**	*BAU*	2,799.4	3,290.0	1,313.6	170.6	101.5	**1,585.7**	1,545.9	182.4	120.1	**1,848.4**
	*HIGH*	11,862.8	20,373.9	5,670.3	661.7	421.5	**6,753.5**	9,859.1	1,010.8	751.7	**11,621.6**
**Egypt**	*BAU*	1,518.8	1,756.1	672.9	85.8	33.2	**791.9**	778.0	90.2	38.4	**906.6**
	*HIGH*	4,334.4	6,868.3	1,920.2	244.8	94.8	**2,259.8**	3,042.8	352.6	150.3	**3,545.7**
**Ghana**	*BAU*	122.7	148.1	81.8	3.2	10.2	**95.2**	98.7	3.5	12.3	**114.5**
	*HIGH*	810.2	1,628.4	540.1	21.0	67.5	**628.6**	1,085.6	38.4	135.7	**1,259.7**
**Kenya**	*BAU*	70.0	80.4	31.0	11.3	6.5	**48.8**	35.7	11.8	7.4	**54.9**
	*HIGH*	70.0	94.5	31.0	11.3	6.5	**48.8**	41.9	13.9	8.7	**64.5**
**Malawi**	*BAU*	7.7	9.3	4.6	0.3	0.2	**5.1**	5.6	0.3	0.2	**6.1**
	*HIGH*	7.7	11.0	4.6	0.3	0.2	**5.1**	6.6	0.4	0.2	**7.2**
**Nigeria**	*BAU*	627.6	741.7	330.2	50.3	39.5	**420.0**	390.2	54.0	46.6	**490.8**
	*HIGH*	706.0	998.4	371.4	56.6	44.4	**472.4**	525.3	72.7	62.8	**660.8**
**Tanzania**	*BAU*	14.5	16.5	8.9	1.6	2.0	**12.5**	10.2	1.6	2.3	**14.1**
	*HIGH*	14.5	19.2	8.9	1.6	2.0	**12.5**	11.8	1.9	2.6	**16.3**
**Uganda**	*BAU*	266.7	306.6	113.4	5.3	2.4	**121.1**	130.4	5.5	2.7	**138.6**
	*HIGH*	270.9	366.3	115.2	5.4	2.4	**123.0**	155.7	6.6	3.2	**165.5**
**Zambia**	*BAU*	51.5	60.9	14.5	5.6	3.2	**23.3**	17.1	6.0	3.8	**26.9**
	*HIGH*	109.0	184.3	30.7	11.8	6.9	**49.4**	51.9	18.1	11.6	**81.6**

All value costs are in millions of constant 2010 US$.

Under the *HIGH* scenarios, aquaculture production values in Africa are projected to reach US$ 11.9 billion in 2030 and US$ 20.4 billion in 2050 (Table 7). To maintain the aquaculture growth rate as projected in the *HIGH* scenario, investment costs in the three key variable costs of feed, labor, and seed need to increase to US$ 6.8 billion by 2030 and US$ 11.6 billion by 2050. These key investment costs will be invested by producers, including private aquaculture enterprises and farmers at different production scales. Similar to the *BAU* scenario, feed cost is the main component, accounting for more than 80% (Fig 4). Investing in aquaculture feed is critical to achieving the aquaculture production outputs by 2030 and 2050 projected in the *HIGH* scenario.

Our post-model estimation (Table 7) suggests that uneven distribution of future aquaculture production values and required investment costs will remain under both the *BAU* and *HIGH* scenarios. Under the *BAU*, the eight countries included in this study are projected to account for 96% of Africa’s aquaculture production values in 2030 and only slightly reduce to 95% by 2050. A similar pattern is observed for investment costs of the key variable inputs required to achieve aquaculture projection output levels. The top four African aquaculture producers–namely Egypt, Nigeria, Uganda, and Ghana–account for 90% of the production values while the group of Kenya, Zambia, Tanzania, and Malawi was projected to account for 5% throughout 2030 and 2050.

## Discussion

One of Africa’s biggest development challenges is to meet the nutrition needs, within sustainable limits of 2.4 billion women, men and children by 2050 [91]. Experiences in Asia and other regions show that aquatic foods, capture fisheries, and aquaculture systems [92] offer important nutritional and sustainability values, in some cases outperforming nutritional qualities of dietary supplements [93] and a relatively lower environmental footprint than animal-source foods [94, 95]. However, the role of fisheries, aquaculture and aquatic foods in the transformation of food systems has remained relatively overlooked due to the lack of scientific data, metrics, and evidence to inform donors, governments, and private investors in decision making and investment planning [96]. Using a rigorous partial equilibrium economic modeling tool, the IMPACT fish model, we generate future fish supply-demand projections in Africa to 2050 under the *BAU* and *HIGH* scenarios. Fish supply projections are then used to extrapolate future direct and indirect employment and investment costs needed to achieve the projected output levels.

There is a global concern that current food systems are ill-equipped to deliver nutritious food, a challenge that will be exacerbated as demands of burgeoning populations and wealth will outpace supplies. In practice, limited supplies of quality food will affect different people to different degrees based on their economic status, geography, and gender, with women of reproductive age and children under the age of five being most vulnerable to nutrient deficiencies [97]. Our *BAU* scenario projection shows that per capita fish consumption in Africa will gradually drop from 10.0 kg/person/year (about half the global and Asian fish intake) in 2015 to 7.7 kg/person/year in 2050. The drop in fish consumption we see in our *BAU* scenario is a result of population growth outpacing growth in the fish sector. Decreasing per capita fish consumption is also the outcome of modest GDP growth. Lower economic growth will constrain governments’ and private sector’s ability to invest in supply infrastructure, technology, and management systems that might otherwise boost supplies. In the optimistic *HIGH* scenario, with GDP growth at 4.8% per year to 2050, per capita fish consumption is projected to increase from 10.0 kg/year in 2015 to 14.0 kg/year in 2050. Investments in sustainable fisheries management and aquaculture will boost total domestic production to 34.8 million tonnes in 2050, of which the share of capture fisheries in total fisheries production in Africa will decline from 82.7% in 2015 to 46.0% in 2050. These results show that there is potential to sustain capture fisheries and expand aquaculture to meet the growing demand for fish in Africa. This needs a sound enabling macro-environment, particularly moderate to high economic growth to stimulate fish demand increase and sustained investment from farmers, investors and governments to transform Africa’s capture fisheries and aquaculture into sustainable, productive, nutrition-sensitive and inclusive aquatic food systems.

Youth employment, and the future employment of current youth, is a growing opportunity and concern globally, and aquaculture and fisheries offer possible but evolving opportunities. With rapid population growth and a young population (60% of the African population below the age of 25), it is expected that 11 million young people will enter the job market in Sub-Saharan Africa every year, while only about 3 million new jobs are created annually on the continent [98–100]. Both capture fisheries and aquaculture are important sources of employment in Africa, particularly for smallholders and value chain actors in rural areas [100]. Creating jobs in rural areas at a large-scale is critical to address these unemployment issues and income generation in Africa. Our study results show that under the *BAU* scenario, with slow aquaculture growth and almost stagnant capture fisheries, Africa’s fish sector is projected to provide 22 million direct and indirect jobs by 2050. However, with the *HIGH* scenario, 58 million people will be directly and indirectly employed in fisheries and aquaculture sectors, representing 2.4% of the total projected population in Africa in 2050. The projection results indicate that growth in Africa’s fish sector will create considerable employment and has the potential to generate significant income growth and facilitate inclusive value chain development to address development barriers faced by Africa. About 60 million people (14% of whom are women) were engaged in the primary sector to produce 179 million tonnes of fish globally in 2018 [101], implying a global average labor productivity of 3.0 tonnes/worker. Our projection estimated that the overall African labor productivity of direct employment in both capture fisheries and aquaculture is 2.0 tonnes/worker, slightly lower than the world average. Furthermore, employment in the fish sector in Africa will continue to be dominated by small-scale fisheries, with lower labor productivity compared to aquaculture (1.7 tonnes/worker vs. 6.3 tonnes/worker). This result highlights the importance of sustainable capture fisheries management to generate employment opportunities and provide income for the portion of the African population depending on artisanal fishing. In order to achieve the desired sustainability transformation, public policy leadership and private sector technological innovation will be required [102].

Our projection results show that strong aquaculture growth has a high potential to generate income and jobs for rural communities in Africa. Under the *HIGH* scenario, aquaculture production in Africa is projected to reach 18.8 million tonnes, generating a revenue of US$ 20.4 billion in 2050. Projected farm-gate investment costs of three key aquaculture inputs (feed, labor, and fish seed) will reach US$ 11.6 billion in 2050. It is essential to highlight that these investments can be mobilized from farmers, private sector investors and enterprises, suggesting dynamic opportunities for market-led aquaculture business development. Given that feed accounts for a major share of aquaculture production cost, this suggests that there will be bright prospects for investing in the aquaculture feed industry in Africa. It will be essential to have more supportive policies and regulations to serve as an entry point for the private sector on more inclusive ways to engage smallholders in the fish value chains.

This study provides useful insights on how aquatic foods, fisheries and aquaculture systems in Africa might evolve into the future under complex and dynamic interactions of structural changes, technological progress, income growth, and urbanization in a climate crisis. The study findings also allow drawing policy implications of different impact pathways, drivers and interventions to enhance aquatic food systems’ contributions to sustainable development goals in Africa. As documented in a previous report [103], these results could be the practical usage by a wide range of stakeholders from international organizations, academic and national government. Notwithstanding these contributions, our study has several limitations due to data gaps. First, in aquaculture investment cost extrapolation, we do not estimate the required investments for farm or value chain infrastructure. Second, we are unable to project investment costs needed for capture fisheries monitoring, management and capacity building, which mostly come from public funding and development and conservation funding. Future follow-up studies should investigate aquaculture infrastructure cost requirements and investment costs for capture fisheries management in Africa. Third, our extrapolation of outcomes focuses only on employment opportunities. Further research is needed to extend the post-model analysis to examine implications on other outcome areas such as gender equity, nutrition and environmental sustainability associated with different future projected trends. Effective and efficient use of data collection tools for gender and youth assessment needs to be embedded in a future inclusive development process. Fourth, our estimation of investment costs does not include public investment in infrastructure, human capital and research capacity needed to create macro and micro enabling environment for aquaculture and fisheries sector performance. Finally, aquatic foods are relatively new in the realm of foresight modeling tools compared to crops and livestock, and further advancement of fish foresight modeling tools is essential to improve the quality of modeling projections and incorporating these outcomes in future analyses. Given the high diversity in wild-caught and cultured fish species in Africa and worldwide, the current IMPACT model is highly aggregated with sixteen fish categories on the supply side and nine categories on the demand side. The model is calibrated using data in 2000 as a base year. This is quite out-of-date given that fisheries and aquaculture are complex and very dynamic, experiencing rapid growth over the last two decades. The IMPACT fish model uses generic assumptions to obtain parameters for specifying the fish sector equations, whereas fish and aquatic food systems are highly heterogeneous and complex. There are numerous fish types, classification schemes, and production methods. It is necessary to conduct disaggregated modeling studies for specific fish types to capture the diversity of trends within specific sub-sectors. Follow-up foresight modeling analysis and projection could address these disaggregation and complexity issues.

## Conclusions

Our current food systems face severe challenges in achieving equitable access to healthy, nutritious food, maintaining environmental sustainability, and building resilience to shocks. Fish and aquatic foods offer significant potential in the transformation of food systems toward healthy and sustainable diets, sustaining livelihoods, and generating income. The fish sectors are important for employment creation in Africa, yet, their role has been overlooked, resulting in insufficient investment to support the sector growth and sustainable system transformation to meet the increasing demand for fish. This study provides insights into future fish supply and demand projections in Africa under the *BAU* and *HIGH* scenarios and provides first estimates of employment generated and the necessary input cost investments required to secure projected fish supplies in 2030 and 2050. The study suffers limitations that should be addressed in the future. Nonetheless, this is a key and first preliminary analysis to look at macro-level employment and investment scenarios of fish sectors in Africa.

## References

[1] Chan CY, Tran N, Pethiyagoda S, Crissman CC, Sulser TB, Phillips MJ. Prospects and challenges of fish for food security in Africa. Glob Food Secur. 2019; 20: 17–25. 10.1016/j.gfs.2018.12.002

[2] Springmann M, Clark M, Mason-D’Croz D, Wiebe K, Bodirsky BL, Lassaletta L, et al. Options for keeping the food system within environmental limits. Nature. 2018. doi: 10.1038/s41586-018-0594-0 30305731

[3] Troell M, Naylor RL, Metian M, Beveridge M, Tyedmers PH, Folke C, et al. Does aquaculture add resilience to the global food system? Proc Natl Acad Sci USA. 2014; 111(37): 13257–13263. doi: 10.1073/pnas.1404067111 25136111 PMC4169979

[4] UN. Department of Economic and Social Affairs, Population Division. World Population Prospects 2019. 2021. Available from: https://population.un.org/wpp

[5] African Development Bank. Africa in Fifty Year’s Time: The road towards inclusive growth. Tunis, Tunisia. 2011:76. Available from: https://www.afdb.org/fileadmin/uploads/afdb/Documents/Publications/Africa%20in%2050%20Years%20Time.pdf

[6] Obiero K, Meulenbroek P, Drexler S, Dagne A, Akoll P, Odong R, et al. The Contribution of Fish to Food and Nutrition Security in Eastern Africa: Emerging Trends and Future Outlooks. Sustain. 2019; 11(6). 10.3390/su11061636

[7] O’Meara L, Cohen PJ, Simmance F, Marinda P, Nagoli J, Teoh SJ, et al. Inland fisheries critical for the diet quality of young children in sub-Saharan Africa. Glob Food Secur. 2021; 28: 100483. 10.1016/j.gfs.2020.100483

[8] Tran N, Chu L, Chan CY, Genschick S, Phillips MJ, Kefi AS. Fish supply and demand for food security in Sub-Saharan Africa: An analysis of the Zambian fish sector. Mar Policy. 2019; 99: 343–350. 10.1016/j.marpol.2018.11.009

[9] Willett W, Rockström J, Loken B, Springmann M, Lang T, Vermeulen S, et al. Food in the Anthropocene: the EAT-Lancet Commission on healthy diets from sustainable food systems. Lancet. 2019; 393(10170): 447–492. doi: 10.1016/S0140-6736(18)31788-4 30660336

[10] HLPE. Sustainable fisheries and aquaculture for food security and nutrition. A report by the high level panel of experts on food security and nutrition of the committee on world food security Rome: FAO. 2014. Available from: http://www.fao.org/3/i3844e/i3844e.pdf

[11] Funge-Smith SJ. Review of the state of world fishery resources: inland fisheries. FAO Fisheries and Aquaculture Circular No. C942 Rev.3, Rome. 397 pp. 2018. Available from: http://www.fao.org/3/CA0388EN/ca0388en.pdf

[12] FAO. Fishery and Aquaculture Statistics. Global aquaculture production 1950–2019 (FishstatJ). In: FAO Fisheries and Aquaculture Department [online]. Rome. 2021. Available from: www.fao.org/fishery/statistics/software/fishstatj/en

[13] Naylor RL. Oil crops, aquaculture, and the rising role of demand: A fresh perspective on food security. Glob food secur. 2016; 11: 17–25. 10.1016/j.gfs.2016.05.001

[14] Thurstan RH, Roberts CM. The past and future of fish consumption: Can supplies meet healthy eating recommendations? Mar Pollut Bull. 2014; 89(1–2): 5–11. doi: 10.1016/j.marpolbul.2014.09.016 25261177

[15] Victora CG, Christian P, Vidaletti LP, Gatica-Domínguez G, Menon P, Black RE. Revisiting maternal and child undernutrition in low-income and middle-income countries: variable progress towards an unfinished agenda. Lancet. 2021; 397(10282): 1388–1399. doi: 10.1016/S0140-6736(21)00394-9 33691094 PMC7613170

[16] Béné C, Lawton R, Allison EH. “Trade Matters in the Fight Against Poverty”: Narratives, Perceptions, and (Lack of) Evidence in the Case of Fish Trade in Africa. World Dev. 2010; 38(7): 933–954. 10.1016/j.worlddev.2009.12.010

[17] Chan CY, Tran N, Dao CD, Sulser TB, Phillips MJ, Batka M, et al. Fish to 2050 in the ASEAN region. Penang, Malaysia: WorldFish and Washington DC, USA: International Food Policy Research Institute (IFPRI) Working Paper. 2017; 2017–01. Available from: http://pubs.iclarm.net/resource_centre/2017-01.pdf

[18] Hope KR. Climate change and poverty in Africa. Inter J Sustain Dev & World Ecology. 2009; 16(6): 451–461. 10.1080/13504500903354424

[19] Barange M, Cheung WWL, Merino G, Perry RI. Modelling the potential impacts of climate change and human activities on the sustainability of marine resources. Curr Opi Environ Sustain. 2010; 2(5–6): 326–333. 10.1016/j.cosust.2010.10.002

[20] Cheung WWL, Lam VWY, Sarmiento JL, Kearney K, Watson REG, Zeller D, et al. Large-scale redistribution of maximum fisheries catch potential in the global ocean under climate change. Glob Change Bio. 2010; 16(1): 24–35. 10.1111/j.1365-2486.2009.01995.x

[21] Lam VWY, Cheung WWL, Swartz W, Sumaila UR. Climate change impacts on fisheries in West Africa: implications for economic, food and nutritional security. Afr J Mar Sci. 2012; 34(1): 103–117. 10.2989/1814232x.2012.673294

[22] Hollowed AB, Barange M, Beamish RJ, Brander K, Cochrane K, Drinkwater K, et al. Projected impacts of climate change on marine fish and fisheries. ICES J Mar Sci. 2013; 70(5): 1023–1037. 10.1093/icesjms/fst081

[23] IPCC. Climate change 2007: Impacts, adaptations and vulnerability. In Parry M. L., Canzian O. F., Palutikof J. P., van der Linden P. J., Paul J.& Hanson C.(Eds.), Contribution of working group II to the 4th assessment report of the Intergovernmental Panel on Climate Change (IPCC). Cambridge: Cambridge University Press. 2007.

[24] IPCC. Climate change: impacts, adaptation and vulnerability. In Working group II contribution to the intergovernmental panel on climate change. Summary for policy makers. IPCC Secretariat, Geneva, Switzerland. 2007. Available from: https://www.ipcc.ch/working-group/wg2/

[25] Belhabib D, Cheung WWL, Kroodsma D, Lam VWY, Underwood PJ, Virdin J. Catching industrial fishing incursions into inshore waters of Africa from space. Fish Fish. 2019; 21(2): 379–392. 10.1111/faf.12436

[26] Belton B, Rosen L, Middleton L, Ghazali S, Mamun A-A, Shieh J, et al. COVID-19 impacts and adaptations in Asia and Africa’s aquatic food value chains. Mar Policy. 2021; 129: 104523. doi: 10.1016/j.marpol.2021.104523 34744258 PMC8564473

[27] NEPAD. Planning and Coordinating Agency, African Union Inter-African Bureau for Animal Resources. The Pan-African Fisheries and Aquaculture Policy Framework and Reform Strategy: Promoting Inter-Regional Fish Trade. NPCA, AU-IBAR, Midrand, South Africa. 2016.

[28] Delgado CL, Wada N, Rosegrant MW, Meijer S, Ahmed M. Fish to 2020: Supply and Demand in Changing Global Markets: WorldFish Center Technical Report 62. Washington, DC: International Food Policy Research Institute; 2003. Available from: http://pubs.iclarm.net/resource_centre/WF_356.pdf

[29] World Bank. Fish to 2030: Prospect for Fisheries and Aquaculture. Washington, DC: World Bank. 2013. Available from: http://documents1.worldbank.org/curated/en/458631468152376668/pdf/831770WP0P11260ES003000Fish0to02030.pdf

[30] IFPRI. Repository for IFPRI’s IMPACT model. 2020. Available from: https://github.com/IFPRI/IMPACT

[31] FAO. Fishery and Aquaculture Statistics. Food Balance Sheets 1961–2018 (FishstatJ). In: FAO Fisheries and Aquaculture Department [online]. Rome. 2021. Available from: www.fao.org/fishery/statistics/software/fishstatj/en

[32] FAO. Low-Income Food-Deficit Countries (LIFDCs)-List updated June 2021. 2021. Available from: https://www.fao.org/countryprofiles/lifdc/en

[33] World Bank. World Bank Open Data. 2021. Available from: https://data.worldbank.org

[34] FAO. FAOSTAT statistics database. Food balance sheets. 2021. Available from: http://www.fao.org/faostat/en/#data/FBS

[35] O’Neill BC, Kriegler E, Ebi KL, Kemp-Benedict E, Riahi K, Rothman DS, et al. The roads ahead: Narratives for shared socioeconomic pathways describing world futures in the 21st century. Glob Environ Change. 2017; 42: 169–180. 10.1016/j.gloenvcha.2015.01.004

[36] Kolding J, Zwieten PAMv, Mosepele K. "Where there is water there is fish". Small-scale inland fisheries in Africa: dynamics and importance. In: Tvedt T, Oestigaard T, editors. A History of Water Vol 3 Water and Food: From hunter-gatherers to global production in Africa. London: I.B. Tauris; 2015.

[37] FAO. The State of World Fisheries and Aquaculture 2018—Meeting the sustainable development goals. Rome. 2018. Available from: http://www.fao.org/3/i9540en/i9540en.pdf

[38] Valderrama D, Hishamunda N, Zhou X. Estimating Employment in World Aquaculture. FAO Aquaculture Newsletter No 45. 2011: 24–25. Available from: http://www.fao.org/3/al363e/al363e12.pdf

[39] Aheto DW, Acheampong E, Odoi JO. Are small-scale freshwater aquaculture farms in coastal areas of Ghana economically profitable? Aqua Inter. 2019; 27(3): 785–805. 10.1007/s10499-019-00363-9

[40] Anker R, Anker M. Living Wage for rural Malawi with Focus on Tea Growing area of Southern Malawi. Fairtrade International, Sustainable Agriculture Network/Rainforest Alliance and UTZ Certified, 70. 2014.

[41] Dey MM, Paraguas FJ, Kambewa P, Pemsl DE. The impact of integrated aquaculture-agriculture on small-scale farms in Southern Malawi. Agr Econ. 2010; 41(1): 67–79. 10.1111/j.1574-0862.2009.00426.x

[42] Hasan MR, Hecht T, De Silva SS, Tacon AGJ. Study and analysis of feeds and fertilizers for sustainable aquaculture development. FAO Fisheries Technical Paper No 497 Rome, FAO 2007. 2007:510p. Available from: http://www.fao.org/3/a1444e/a1444e00.htm

[43] Hyuha TS, Egna H, Ekere W. Social and economic performance of tilapia farming in Uganda. In Cai J., Quagrainie K. K. & Hishamunda N., eds. Social and economic performance of tilapia farming in Africa, pp.127–144. FAO Fisheries and Aquaculture Circular No 1130 Rome, Italy. 2017. Available from: http://www.fao.org/3/a-i7258e.pdf

[44] Jacobi N. Examining the Potential of Fish Farming to Improve the Livelihoods of Farmers in the Lake Victoria Region, Kenya—Assessing Impacts of Governmental Support. Faculty of Business and Science University of Akureyri, University Centre of the Westfjords, Master of Resource Management: Coastal and Marine Management Ísafjörður, May 2013. 2013. Available from: https://aquadocs.org/bitstream/handle/1834/6854/ktf0232.pdf?sequence=1

[45] Kaliba AR, Osewe KO, Senkondo EM, Mnembuka BV, Quagrainie KK. Economic Analysis of Nile Tilapia (Oreochromis niloticus) Production in Tanzania. J World Aqua Soc. 2006; 37(4): 464–473. 10.1111/j.1749-7345.2006.00059.x

[46] Kaminski AM, Genschick S, Kefi AS, Kruijssen F. Commercialization and upgrading in the aquaculture value chain in Zambia. Aqua. 2018; 493: 355–364. 10.1016/j.aquaculture.2017.12.010

[47] Kofi F, Nunoo E, Asamoah EK, Osei-asare YB. Economics of aquaculture production: a case study of pond and pen culture in southern Ghana. Aqua Res. 2014; 45: 675–688. doi: 10.1111/are.12003

[48] Kyelu A. Analysis of socio-economic and environmental effects of urban fish farming in Dar es Salaam, Tanzania. Sokoine University of Agriculture. 2016. Available from: http://www.suaire.sua.ac.tz/handle/123456789/1428

[49] Masike S, Liwenga E. Cost Benefit Analysis of the Community Livelihoods Improvement Initiatives in Tanzania. Pro-poor Economic Growth and Environmentally Sustainable Development Project. 2017. Available from: https://pea4sdgs.org/sites/default/files/2020-09/Cost%20Benefit%20Analysis%20of%20the%20Community%20Livelihoods%20Improvement%20Initiatives%20in%20Tanzania_2.pdf

[50] Mwango J, Kefi AS, Manda EC, Chijoka M, Chimba N. Status of aquaculture in the Copperbelt and North-western provinces of Zambia. Republic of Zambia Ministry of Fisheries and Livestock. 2016: 1–68. Available from: https://www.academia.edu/30644672/STATUS_OF_AQUACULTURE_IN_THE_COPPERBELT_AND_NORTH_WESTERN_PROVINCES_OF_ZAMBIA

[51] Nasr-Allah A, Macfadyen G, Dickson M, Al–Kenawy D, Fathi M, El-Naggar G. Value Chain Analysis of the Egyptian Aquaculture Sector. IIFET 2012 Tanzania Proceedings. 2012. 10.13140/2.1.3709.2808

[52] Nyandat B, Owiti GO. Aquaculture needs assessment mission report. Report/Rapport: SF-FAO/2013/24. September/Septembre 2013. FAO-SmartFish Programme of the Indian Ocean Commission, Ebene, Mauritius. 2013. Available from: http://www.fao.org/3/az041e/az041e.pdf

[53] Safina N, Gertrude A, Lawrance O, Ronald W, Alphonse C, Samuel O, et al. Profitability and Viability Analysis of Aquaculture Production in Central Uganda: A Case of Urban and Peri-Urban Areas. Asian J Agr Ext, Econ & Soc. 2018; 22(4): 1–11. 10.9734/ajaees/2018/37721

[54] Tran N, Crissman C, Chijere A, Hong MC, Teoh SJ, Valdivia RO. Ex-ante assessment of integrated aquaculture-agriculture adoption and impact in Southern Malawi. Working paper: AAS-2013-03, CGIAR. 2013. Available from: http://pubs.iclarm.net/resource_centre/WF_3540.pdf

[55] Kleih U, Linton J, Marr A, Mactaggart M, Naziri D, Orchard JE. Financial services for small and medium-scale aquaculture and fisheries producers. Mar Policy. 2013; 37: 106–114. 10.1016/j.marpol.2012.04.006

[56] Amankwah A, Quagrainie KK. Aquaculture feed technology adoption and smallholder household welfare in Ghana. J World Aqua Soc. 2018: 1–15. 10.1111/jwas.12544

[57] Amenyogbe E, Chen G, Wang Z, Lin M, Lu X, Atujona D, et al. A Review of Ghana’s Aquaculture Industry. J Aqua Res & Dev. 2018; 09(08). 10.4172/2155-9546.1000545

[58] Anane-Taabeah G, Quagrainie K, Amisah S. Assessment of farmed tilapia value chain in Ghana. Aqua Int. 2015; 24(4): 903–919. 10.1007/s10499-015-9960-1

[59] FAO. Fishery and Aquaculture Country Profiles—The Republic of Ghana. FAO Fisheries & Aquaculture. 2016. Available from: http://www.fao.org/fi/oldsite/FCP/en/GHA/profile.htm

[60] Karikari AY. Assessment of Environmental Impacts of Cage Aquaculture on Lake Volta of Ghana. Kwame Nkrumah University of Science and Technology. 2016. Available from: http://129.122.16.11/bitstream/123456789/9976/1/FINAL%20THESIS.pdf

[61] Kassam L, Dorward A. A comparative assessment of the poverty impacts of pond and cage aquaculture in Ghana. Aqua. 2017; 470: 110–122. 10.1016/j.aquaculture.2016.12.017

[62] Mensah ETD, Dankwa HR, Torben LL, Asmah R, Campion BB, Edziyie R. Effects of seasonal and environmental changes on aquaculture production in tropical Lake Volta, Ghana. Aqua Int. 2018; 26(6): 1387–1400. 10.1007/s10499-018-0294-7

[63] Nunoo FKE, Asamoah EK, Osei-Asare YB. Economics of aquaculture production: a case study of pond and pen culture in southern Ghana. Aqua Res. 2014; 45(4): 675–688. 10.1111/are.12003

[64] Ofori JK, Abban EK, Karikari AY, Brummett RE. Production Parameters and Economics of Small-Scale Tilapia Cage Aquaculture in the Volta Lake, Ghana. J App Aqua. 2010; 22(4): 337–351. 10.1080/10454438.2010.527591

[65] Consulting L. Market Study of the Aquaculture Market in Kenya—Kenya Market-led Aquaculture Programme (KMAP). 2016: 1–75. Available from: https://www.farmafrica.org/downloads/study-of-the-kenyan-aquaculture-market.pdf

[66] KMFRI. Kenya Aquaculture Brief 2017: Status, Trends, Challenges and Future Outlook. Kenya Marine and Fisheries Research Institute. 2017. Available from: https://www.kmfri.co.ke/images/pdf/Kenya_Aquaculture_Brief_2017.pdf

[67] Kundu R, Muchiri M, Njiru M, Nyamweya C. Effect of Social and Economic Drivers on Success of Small Scale Fish farming in Western Kenya. Afr J Trop Hydrobiology Fish. 2016(14): 29–44.

[68] Munguti JM, Kim J-D, Ogello EO. An Overview of Kenyan Aquaculture: Current Status, Challenges, and Opportunities for Future Development. Fish aqua sci. 2014; 17(1): 1–11. 10.5657/fas.2014.0001

[69] Ndanga LZB, Quagrainie KK, Dennis JH. Economically feasible options for increased women participation in Kenyan aquaculture value chain. Aqua. 2013; 414–415: 183–190. 10.1016/j.aquaculture.2013.08.012

[70] Neira I, Engle CR, Ngugi C. Economic and Risk Analysis of Tilapia Production in Kenya. J App Aqua. 2009; 21(2): 73–95. 10.1080/10454430902892842

[71] Opiyo MA, Marijani E, Muendo P, Odede R, Leschen W, Charo-Karisa H. A review of aquaculture production and health management practices of farmed fish in Kenya. Int J Vet Sci Med. 2018; 6(2): 141–148. doi: 10.1016/j.ijvsm.2018.07.001 30564588 PMC6286394

[72] Shikoli WP, Mukoya-Wangia S, Joyce G M, Jesse T N, Nicholas S. Enabling environment for fish farming as an alternative livelihood in Makueni, Kenya. Int J Fish Aqua Studies. 2017; 5(2): 679–683. Available from: https://www.fisheriesjournal.com/archives/2017/vol5issue2/PartI/5-1-11-257.pdf

[73] Wambua MM. A cost–benefit analysis of the fish farming enterprise productivity program project in Kenya. The case of implementation of the aquaculture development component in Meru County. United Nations University Fisheries Training Programme, Iceland [final project]. 2018. Available from: http://www.unuftp.is/static/fellows/document/moses15prf.pdf

[74] Limuwa M, Singini W, Storebakken T. Is Fish Farming an Illusion for Lake Malawi Riparian Communities under Environmental Changes? Sustain. 2018; 10(5). doi: 10.3390/su10051453

[75] Mussa H, Kaunda E, Banda L. Economic analysis of small-scale fish farming in Bunda, Lilongwe, Malawi. RUFORUM Working Document Series. 2016; 14(2): 855–860. Availabe from: https://www.cabdirect.org/cabdirect/abstract/20193088337

[76] Phiri F, Yuan X. Economic Profitability of Tilapia Production in Malawi and China. J Aqua Res Dev. 2018; 09(05). 10.4172/2155-9546.1000535

[77] Oluwatobi AA, Mutalib HA, Adeniyi TK, Olabode JO, Adeyemi A. Possible Aquaculture Development in Nigeria: Evidence for Commercial Prospects. J Agr Sci Tech B. 2017; 7(3): 194–205. 10.17265/2161-6264/2017.03.007

[78] Ayinla OA. Analysis of feeds and fertilizers for sustainable aquaculture development in Nigeria. In: Hasan MR, Hecht T, Silva SSD, Tacon AGJ, editors. Study and analysis of feeds and fertilizers for sustainable aquaculture development FAO Fisheries Technical Paper No 497. Rome: FAO; 2007. p. 453–70.

[79] Dauda AB, Natrah I, Karim M, Kamarudin MS, Bichi AuH. African Catfish Aquaculture in Malaysia and Nigeria: Status, Trends and Prospects. Fish Aqua J. 2018; 09(01). 10.4172/2150-3508.1000237

[80] Emmanuel O, Chinenye A, Oluwatobi A, Peter K. Review of Aquaculture Production and Management in Nigeria. Amer J Exper Agr. 2014; 4(10): 1137–1151. Available from: http://eprints.covenantuniversity.edu.ng/3561/1/AJEA%20final%20publication.pdf

[81] Igoni-Egweke QN. Analysis of Value Addition in Commercial Catfish (Clarias gariepinus Heterobranchus spp.) Production in Rivers State, Nigeria. Federal University of Technology Owerri. 2018. Available from: https://africantheses.org/abstracts/4739-197615

[82] FAO. National Aquaculture Sector Overview—United Republic of Tanzania. FAO Fisheries & Aquaculture. 2016. Available from: http://www.fao.org/fishery/countrysector/naso_tanzania/en

[83] Ibengwe L, Sobo F. The Value of Tanzania Fisheries and Aquaculture: Assessment of the Contribution of the Sector to Gross Domestic Product. In: Taylor WW, Bartley DM, Goddard CI, Leonard NJ, Welcomme R, editors. Freshwater, fish and the future: proceedings of the global cross-sectoral conference. Rome; East Lansing; Bethesda, Maryland: Food and Agriculture Organization of the United Nations; Michigan State University; American Fisheries Society; 2011.

[84] Kleih U, Kishe M, Yunus KB. Financial Services for SME Fisheries Producers in Tanzania. 2010. Available from: http://africanfisheriesinvestment.org/files/casestudies/casestudy-tanzania.pdf

[85] Ministry of Agriculture Livestock and Fisheries. The Tanzanian Fisheries Sector—Challenges and opportunities. United Republic of Tanzania. 2016. Available from: https://www.wiomsa.org/download/the-tanzanian-fisheries-sectors-challenges-and-opportunities/

[86] Rukanda JJ. Evaluation of aquaculture development in Tanzania. United Nations University Fisheries Training Programme, Iceland. 2018. Available from: https://www.grocentre.is/static/gro/publication/353/document/janeth16aprf.pdf

[87] Stutzman E, Molnar J, Atukunda G, Walakira J. Understanding the Role of Fish Farmer Associations as Intermediaries for the Commercialization of Aquaculture in Uganda. Fish Aqua J. 2017; 08(03). 10.4172/2150-3508.1000214

[88] Genschick S, Kaminski A, Cole S, Tran N, Chimatiro S, Lundeba M. Toward more inclusive and sustainable development of Zambian aquaculture. Penang, Malaysia: CGIAR Research Program on Fish Agri-Food Systems Program Brief: FISH-2017-07. 2017. Available from: https://hdl.handle.net/20.500.12348/16

[89] Musuka CG, Musonda FF. Current and future prospects of commercial fish farming in Zambia. Aquaculture, Aquarium, Conservation & Legislation International Journal of the Bioflux Society. 2012; 5(1): 79–87. Available from: http://bioflux.com.ro/docs/AACL_5.2.5.pdf

[90] Namonje-Kapembwa T, Samboko P. Assessing the Profitability of Small-Scale Aquaculture Fish Production in Zambia. Working Paper 123 Indaba Agricultural Policy Research Institute (IAPRI). 2017. Available from: https://pdf.usaid.gov/pdf_docs/PA00SZ57.pdf

[91] McDermott J, Wyatt AJ. The role of pulses in sustainable and healthy food systems. Ann N Y Acad Sci. 2017; 1392(1): 30–42. doi: 10.1111/nyas.13319 28319656

[92] Thilsted SH, Thorne-Lyman A, Webb P, Bogard JR, Subasinghe R, Phillips MJ, et al. Sustaining healthy diets: The role of capture fisheries and aquaculture for improving nutrition in the post-2015 era. Food Policy. 2016; 61: 126–131. 10.1016/j.foodpol.2016.02.005

[93] Byrd KA, Pincus L, Pasqualino MM, Muzofa F, Cole SM. Dried small fish provide nutrient densities important for the first 1000 days. Matern Child Nutr. 2021: e13192. doi: 10.1111/mcn.13192 33942983 PMC8476445

[94] Hallström E, Bergman K, Mifflin K, Parker R, Tyedmers P, Troell M, et al. Combined climate and nutritional performance of seafoods. J Cleaner Prod. 2019; 230: 402–411. https://doi/org/10.1016/j.jclepro.2019.04.229

[95] Troell M, Jonell M, Crona B. Scoping report: The role of seafood for sustainable and healthy diets. The EAT-Lancet Commission report through a blue lens. 2019;266:1–24. Available from: Available from: https://eatforum.org/content/uploads/2019/11/Seafood_Scoping_Report_EAT-Lancet.pdf

[96] Bennett A, Basurto X, Virdin J, Lin X, Betances SJ, Smith MD, et al. Recognize fish as food in policy discourse and development funding. Ambio. 2021; 50(5): 981–989. doi: 10.1007/s13280-020-01451-4 33454882 PMC7811336

[97] Jennings S, Stentiford GD, Leocadio AM, Jeffery KR, Metcalfe JD, Katsiadaki I, et al. Aquatic food security: insights into challenges and solutions from an analysis of interactions between fisheries, aquaculture, food safety, human health, fish and human welfare, economy and environment. Fish Fish. 2016; 17: 893–938. 10.1111/faf.12152

[98] Arulingam I, Nigussie L, Sellamuttu SS, Debevec L. Youth participation in small-scale fisheries, aquaculture and value chains in Africa and the Asia-Pacific. Penang, Malaysia: CGIAR Research Program on Fish Agri-Food Systems. Program Report: FISH-2019-14. 2019. Available from: https://hdl.handle.net/20.500.12348/3937

[99] African Development Bank. Jobs for youth in Africa strategy: Regional ministerial conferences on youth employment and entrepreneurship in Africa. Abidjan, Côte d’Ivoire: African Development Bank Group. 2017. Available from: https://www.afdb.org/fileadmin/uploads/afdb/Documents/Generic-Documents/Ministerial_Conferences_Report_En.pdf

[100] Filmer D, Fox L. Youth Employment in Sub-Saharan Africa. Africa Development Forum, World Bank Washington, DC. 2014. 10.1596/978-1-4648-0107-5

[101] FAO. The State of World Fisheries and Aquaculture 2020. Sustainability in action. Rome. 2020. Available from: 10.4060/ca9229en

[102] Moberg E, Allison EH, Harl HK, Arbow T, Almaraz M, Dixon J, et al. Combined innovations in public policy, the private sector and culture can drive sustainability transitions in food systems. Nature Food. 2021; 2: 282–290. 10.1038/s43016-021-00261-5 37118460

[103] Lowder SK, Regmi A. Independent Review: Assessment of outcomes based on the use of PIM-supported foresight modeling work, 2012–2018. Washington, DC: International Food Policy Research Institute (IFPRI). 2019. 10.2499/p15738coll2.133608

