# Supplementary material for: The future of fish in Africa: Employment and investment opportunities
Source: PLoS One. 2021 Dec 22;16(12):e0261615. doi: 10.1371/journal.pone.0261615 (PMC8694441; doi:10.1371/journal.pone.0261615)

PLOS ONE

The future of fish in Africa: employment and investment opportunities

--Manuscript Draft--

Manuscript Number:	PONE-D-21-16152
Article Type:	Research Article
Full Title:	The future of fish in Africa: employment and investment opportunities
Short Title:	Fish for Future Africa
Corresponding Author:	Nhuong Tran<br>WorldFish<br>Batu Maung, Penang MALAYSIA
Keywords:	Africa; employment; investment; foresight modeling; fisheries; aquaculture
Abstract:	One of the most pressing challenges facing food systems in Africa is ensuring availability of a healthy and sustainable diet to 2.4 billion people by 2050. The continent has struggled with development challenges, particularly chronic food insecurity and pervasive poverty. In Africa's food systems, fish and other aquatic foods play a multifaceted role in generating income, and providing a critical source of essential micronutrients. We applied the International Model for Policy Analysis of Agriculture Commodities and Trade (IMPACT) to explore two Pan-African scenarios for fish sector growth: a business-as-usual (BAU) scenario and a high-growth scenario for capture fisheries and aquaculture with accompanying strong gross domestic product growth (HIGH). Post-model analysis was used to estimate employment and aquaculture investment requirements for the sector in Africa. Africa's fish sector is estimated to support 20.7 million jobs in 2030, growing to 21.6 million by 2050 under the BAU . Approximately 2.6 people will be employed indirectly along fisheries and aquaculture value chains and systems for every person directly employed in the fish production sector. Under the HIGH scenario, total employment in Africa's fish food system will reach 58.0 million jobs, representing 2.4% of total projected population in Africa by 2050. Aquaculture production value is estimated to achieve US$ 3.3 billion and US$ 20.4 billion per year under the BAU and HIGH scenarios by 2050, respectively. Farm-gate investment costs for the three key inputs (fish feeds, farm labor, and fish seed) to achieve the aquaculture volumes projected by 2050 are estimated at US$ 1.8 billion per year under the BAU and US$ 11.6 billion per year under the HIGH scenario. Sustained investments are critical to sustain capture fisheries and support aquaculture growth for food system transformation towards healthier diets.
Order of Authors:	Chin Yee Chan, MS
	Nhuong Tran
	Kai Ching Cheong
	Timothy B Sulser
	Philippa J Cohen
	Keith Wiebe
	Ahmed Mohamed Nasr-Allah
Additional Information:	
Question	Response
Financial Disclosure<br><br>Enter a financial disclosure statement that describes the sources of funding for the work included in this submission. Review the submission guidelines for detailed	NT and CC received funding from the CGIAR Research Programs on Fish Agri-Food Systems (FISH) led by WorldFish (https://fish.cgiar.org); Policies, Institutions, and Markets (PIM) led by International Food Policy Research Institute (IFPRI) (https://pim.cgiar.org); and Climate Change, Agriculture and Food Security (CCAFS) led by Alliance of Bioversity International and International Center for Tropical Agriculture (https://ccafs.cgiar.org). These programs are supported by contributors to

Powered by Editorial Manager® and ProduXion Manager® from Aries Systems Corporation

requirements. View published research articles from *PLOS ONE* for specific examples.<br><br>This statement is required for submission and **will appear in the published article** if the submission is accepted. Please make sure it is accurate.<br><br>**Unfunded studies**<br>Enter: *The author(s) received no specific funding for this work.*<br><br>**Funded studies**<br>Enter a statement with the following details:<br>• Initials of the authors who received each award<br>• Grant numbers awarded to each author<br>• The full name of each funder<br>• URL of each funder website<br>• Did the sponsors or funders play any role in the study design, data collection and analysis, decision to publish, or preparation of the manuscript?<br>• **NO** - Include this sentence at the end of your statement: *The funders had no role in study design, data collection and analysis, decision to publish, or preparation of the manuscript.*<br>• **YES** - Specify the role(s) played.<br><br>* typeset	the CGIAR Trust Fund. The funders had no role in study design, data collection and analysis, decision to publish, or preparation of the manuscript.
**Competing Interests**<br><br>Use the instructions below to enter a competing interest statement for this submission. On behalf of all authors, disclose any competing interests that could be perceived to bias this work—acknowledging all financial support and any other relevant financial or non-financial competing interests.<br><br>This statement is required for submission and **will appear in the published article** if the submission is accepted. Please make sure it is accurate and that any funding sources listed in your Funding Information later in the submission form are also declared in your Financial Disclosure	The authors have declared that no competing interests exist

statement.<br><br>View published research articles from *PLOS ONE* for specific examples.<br><br>**NO authors have competing interests**<br><br>Enter: *The authors have declared that no competing interests exist.*<br><br>**Authors with competing interests**<br><br>Enter competing interest details beginning with this statement:<br><br>*I have read the journal's policy and the authors of this manuscript have the following competing interests: [insert competing interests here]*<br><br>* typeset	
**Ethics Statement**<br><br>Enter an ethics statement for this submission. This statement is required if the study involved:<br><br>• Human participants<br>• Human specimens or tissue<br>• Vertebrate animals or cephalopods<br>• Vertebrate embryos or tissues<br>• Field research<br><br>Write "N/A" if the submission does not require an ethics statement.<br><br>General guidance is provided below. Consult the submission guidelines for detailed instructions. **Make sure that all information entered here is included in the Methods section of the manuscript.**	N/A

**Format for specific study types**<br><br>**Human Subject Research (involving human participants and/or tissue)**<br>• Give the name of the institutional review board or ethics committee that approved the study<br>• Include the approval number and/or a statement indicating approval of this research<br>• Indicate the form of consent obtained (written/oral) or the reason that consent was not obtained (e.g. the data were analyzed anonymously)<br><br>**Animal Research (involving vertebrate animals, embryos or tissues)**<br>• Provide the name of the Institutional Animal Care and Use Committee (IACUC) or other relevant ethics board that reviewed the study protocol, and indicate whether they approved this research or granted a formal waiver of ethical approval<br>• Include an approval number if one was obtained<br>• If the study involved *non-human primates*, add *additional details* about animal welfare and steps taken to ameliorate suffering<br>• If anesthesia, euthanasia, or any kind of animal sacrifice is part of the study, include briefly which substances and/or methods were applied<br><br>**Field Research**<br><br>Include the following details if this study involves the collection of plant, animal, or other materials from a natural setting:<br>• Field permit number<br>• Name of the institution or relevant body that granted permission	
**Data Availability**<br><br>Authors are required to make all data underlying the findings described fully available, without restriction, and from the time of publication. PLOS allows rare exceptions to address legal and ethical concerns. See the PLOS Data Policy and FAQ for detailed information.	Yes - all data are fully available without restriction

A Data Availability Statement describing where the data can be found is required at submission. Your answers to this question constitute the Data Availability Statement and **will be published in the article**, if accepted.<br><br>**Important:** Stating 'data available on request from the author' is not sufficient. If your data are only available upon request, select 'No' for the first question and explain your exceptional situation in the text box.<br><br>Do the authors confirm that all data underlying the findings described in their manuscript are fully available without restriction?	
**Describe where the data may be found in full sentences. If you are copying our sample text, replace any instances of XXX with the appropriate details.**<br><br>• If the data are **held or will be held in a public repository**, include URLs, accession numbers or DOIs. If this information will only be available after acceptance, indicate this by ticking the box below. For example: *All XXX files are available from the XXX database (accession number(s) XXX, XXX.).*<br>• If the data are all contained **within the manuscript and/or Supporting Information files**, enter the following: *All relevant data are within the manuscript and its Supporting Information files.*<br>• If neither of these applies but you are able to provide **details of access elsewhere**, with or without limitations, please do so. For example:<br><br>*Data cannot be shared publicly because of [XXX]. Data are available from the XXX Institutional Data Access / Ethics Committee (contact via XXX) for researchers who meet the criteria for access to confidential data.*<br><br>*The data underlying the results presented in the study are available from (include the name of the third party*	Data are available on GitHub at https://github.com/IFPRI/IMPACT

and contact information or URL).<br>• This text is appropriate if the data are owned by a third party and authors do not have permission to share the data.<br><br>* typeset	
Additional data availability information:	

Powered by Editorial Manager® and ProduXion Manager® from Aries Systems Corporation

Manuscript Click here to access/download;Manuscript;Manuscript.docx

The future of fish in Africa: employment and investment opportunities

Short title: Fish for Future Africa

Authors

Chin Yee Chan[1*#], Nhuong Tran[1*#], Kai Ching Cheong[1], Timothy B Sulser[2], Philippa J Cohen[1], Keith Wiebe[2], Ahmed Mohamed Nasr-Allah[3]

Affiliations

[1]WorldFish, Penang, Malaysia

[2]International Food Policy Research Institute, Washington DC, United States of America

[3]WorldFish, Sharkia, Egypt

*Corresponding author

E-mail: n.tran@cgiar.org (NT)

Email: c.chan@cgiar.org (CC)

[#]These authors contributed equally to this work.

Abstract

One of the most pressing challenges facing food systems in Africa is ensuring availability of a healthy and sustainable diet to 2.4 billion people by 2050. The continent has struggled with development challenges, particularly chronic food insecurity and pervasive poverty. In Africa's food systems, fish and other aquatic foods play a multifaceted role in generating income, and providing a critical source of essential micronutrients. We applied the International Model for Policy Analysis of Agriculture Commodities and Trade (IMPACT) to explore two Pan-African scenarios for fish sector growth: a business-as-usual (*BAU*) scenario and a high-growth scenario for capture fisheries and aquaculture with accompanying strong gross domestic product growth (*HIGH*). Post-model analysis was used to estimate employment and aquaculture investment requirements for the sector in Africa. Africa's fish sector is estimated to support 20.7 million jobs in 2030, growing to 21.6 million by 2050 under the *BAU*. Approximately 2.6 people will be employed indirectly along fisheries and aquaculture value chains and systems for every person directly employed in the fish production sector. Under the *HIGH* scenario, total employment in Africa's fish food system will reach 58.0 million jobs, representing 2.4% of total projected population in Africa by 2050. Aquaculture production value is estimated to achieve US$ 3.3 billion and US$ 20.4 billion per year under the *BAU* and *HIGH* scenarios by 2050, respectively. Farm-gate investment costs for the three key inputs (fish feeds, farm labor, and fish seed) to achieve the aquaculture volumes projected by 2050 are estimated at US$ 1.8 billion per year under the *BAU* and US$ 11.6 billion per year under the *HIGH* scenario. Sustained investments are critical to sustain capture fisheries and support aquaculture growth for food system transformation towards healthier diets.

Keywords

Africa, employment, investment, foresight modeling, fisheries, aquaculture

Introduction

Ensuring that a healthy and sustainable diet is available to 2.4 billion Africans by 2050 is one of the most pressing challenges facing Africa's food systems [1-4]. The continent has struggled with a series of interconnected development challenges, particularly in fighting chronic food insecurity and overcoming pervasive poverty – the two foundational Sustainable Development Goals [5]. In Africa's food systems, fish and other aquatic foods play a multifaceted role as a way of life, generating income, and providing a critical source of essential micronutrients, particularly for women and infants [1, 6-9]. Nevertheless, the current and future values of fish and aquatic foods are often overlooked in development research, policy and investment. It is argued that this oversight means multiple pathways to address malnutrition and food insecurity are underexplored [10].

Fish consumed in Africa are predominantly provided by capture fisheries operating in rivers, large inland lakes and coastal systems [11]. Whereas aquaculture is one of the fastest growing food production sectors globally [12, 13], aquaculture in Africa supplies only 2.7% of the global aquaculture share in 2019, though is growing fast in Africa in response to market demand in the continent maintaining double digit average annual growth rate of aquaculture in the last two decades [12]. Despite this growth, capture fisheries and aquaculture do not supply sufficient fish and there is a significant gap between fish supplies and consumer demand in Africa [1, 8]. Further, the fish supply gap is projected to widen due to a dramatic increase in fish demand, driven by rapid population and income growth, diet transformation resulting from urbanization, and changing consumer preferences [1, 8, 14]. In addition, to these growing demands, unmet nutritional needs persist and increase, particularly for women of reproductive age, children under the age of five and in the first 1000 days of life [15]. Increasing fish supply, reducing waste and loss, supporting intra-regional and international fish trade, and ensuring equitable distribution and access to fish are important strategies to

address some dietary deficiencies and the costly individual and societal consequences [7, 16, 17].

Africa hosts regions that are amongst the most susceptible to global climate change [18]. Climate change projections [19-22] indicate that most of northern and southern Africa will experience high water stress while eastern, central, and western Africa will be subject to increasingly heavy rains and flooding [23, 24]. Changes in precipitation and temperature patterns due to climate change will create further stress in inland lake, river and oceanic ecosystems with ramifications on as such fish supply and the broader wellbeing of actors in the food systems. Climate change is projected to reduce the potential fisheries catch in the Exclusive Economic Zones (EEZs) in Africa [21]. Coupling with climate change impacts, the activities of foreign fishing vessels in African EEZs also be factors impacting the future of fish availability and access in Africa [25]. In sum, there are growing uncertainties and daunting challenges associated with the future of Africa's food systems associated with large-scale drivers that operate outside and within fisheries and aquaculture systems. These challenges, amplified by the unprecedented COVID-19 pandemic have led the governments of African and regional organizations to determine what potential is offered and limits will be experienced through investment in fisheries and aquaculture as priorities for development interventions designed to address food and nutrition security [26, 27].

Public and private sector investment will be critical to secure diverse supplies of fish and other aquatic foods from capture fisheries and aquaculture. Whilst contributing to food and nutrition security in Africa will require four simultaneous strategies (i.e., increasing fish supply, reducing waste and loss, supporting fish trade, and ensuring equitable distribution and access), in this paper we focus on fish supply that could be achieved by increasing production. To date, there are no estimates of investment and potential returns for domestic fish production in Africa. To contribute to policy debates about the future of fish in Africa, we develop two scenarios; *business-as-usual* (*BAU*) and *high capture fisheries/aquaculture with stronger GDP growth* (*HIGH*). We first project future fish supply and demand in Africa to 2050

using the International Model for Policy Analysis of Agriculture Commodities and Trade (IMPACT). Second, we conduct post-model analysis to extrapolate future potential direct (capture fisheries and aquaculture) and indirect employment that would be associated with the *BAU* and *HIGH* scenarios. Finally, we estimate future aquaculture production value and investment costs required to achieve *BAU* and *HIGH* scenarios.

In this study, we employ data and develop projections for eight African nations (i.e., studied countries are Egypt, Ghana, Kenya, Malawi, Nigeria, Tanzania, Uganda, Zambia). These eight countries are home to 40% of Africa's total population but produce over 95% of aquaculture and 30% of capture fisheries production (by volume) in the continent in 2019. About half of fish consumed in Africa is by these eight countries, suggesting slightly higher per capita fish consumption rates than elsewhere in Africa [28]. Among these eight countries, Uganda, Tanzania, Malawi, Kenya, and Ghana are classified by Food and Agriculture Organization (FAO) as low-income food-deficit countries.

Materials and Methods

To provide more comprehensive and consistent outlooks and prospects of fish and aquatic food systems, efforts have been made to integrate fish into foresight modeling of agriculture and livestock commodities. We apply the IMPACT fish model in this study. This module was developed specifically to produce projections of global and regional fish production, consumption, and trade [29, 30]. Previous application of the model *Fish to 2030* [30] reported business-as-usual (*BAU*) scenario used historical data up through 2009. The projection from that model underestimated the 2010–2015 historical trend of capture fisheries and aquaculture production. To address these shortcomings, we re-calibrate the model with the recent dataset and parameters [4, 12, 31, 32]. Specifically, we revisited the productivity growth assumptions of the model, using expert knowledge informed by fisheries and aquaculture specific biophysical and socio-economic factors and fish management and production targets defined

by national governments. We focused on eight African nations. We selected these countries because they are 1) nations projected to face the largest shortfalls in fish supply relative to demands, 2) experience high rates of fish consumption, and 3) are amongst the nations experiencing relatively rapid growth in aquaculture (Table 1). We consulted 76 experts from Egypt, Nigeria, South Africa, Zambia, Tanzania, Ghana, and Kenya during five stakeholder consultation workshops from 2017 to 2019. We sought the input of experts to update and refine the model, explore alternative scenarios, validate projection results, verify the employment and investment dataset, and verify the post-model employment and investment estimation.

Table 1. Contribution of fish to food security in Africa and the world

Indicator	Year	Egypt	Ghana	Kenya	Malawi	Nigeria	Tanzania	Uganda	Zambia	Studied countries	Africa	World
**Demographic and socio-economic status[a]**												
Population (million)	***2020***	102.3	31.1	53.8	19.1	206.1	59.7	45.7	18.4	536.3	1,340.6	7,794.8
Population growth (%)	***2015-2020***	2.1	2.2	2.3	2.7	2.6	3.0	3.7	3.0	2.6	2.5	1.1
Urban population (%)	***2019***	42.7	56.7	27.5	17.2	51.2	34.5	24.4	44.1	41.9	43.0	55.7
GDP per capita (current US$)	***2019***	3,019	2,202	1,817	412	2,230	1,122	794	1,305	1,994	1,867	11,433
GDP growth %	***2009-2019***	4.8	9.9	9.9	2.2	4.4	8.1	3.5	4.3	5.3	4.2	3.8
Undernourishment (%)	***2018***	4.7	6.5	23.0	18.8	12.6	25.0	41.0	45.6	11.9	12.1	8.9
Unemployment (% total labor force)	***2020***	10.4	4.5	3.0	6.0	9.0	2.2	2.4	12.2	7.1	7.8	6.5
	**Year**	**2017**	***2016***	***2015***	***2016***	***2018***	***2017***	***2016***	***2015***			***2017***
Population below US$1.90 a day (%)		3.8	12.7	37.1	69.2	39.1	49.4	41.3	58.7	n.a.	n.a.	9.3
**Contribution of fish to food supply[b]**												
Total fish production (thousand tonnes)	***2019***	2,039	445	144	163	1,115	487	706	136	5,235	12,385	177,834
Share of aquaculture production (%)	***2019***	80.5	11.8	12.9	5.1	26.0	3.4	14.6	28.3	41.4	18.4	48.0
Aquaculture average annual growth (%)	***1999-2019***	10.4	15.6	22.9	14.1	13.8	24.7	30.9	11.7	11.3	11.1	5.2
Capture fisheries average annual growth (%)	***1999-2019***	-0.3	-1.2	-2.4	6.3	3.0	2.1	5.0	1.9	1.6	2.3	0.04
**Contribution of fish to food and nutritional status[c]**												
Fish consumption (kg/capita/year)	***2018***	23.2	24.8	3.0	11.9	8.9	6.8	10.9	11.7	12.1	10.3	20.2
Fish protein (g/capita/day)	***2018***	6.6	8.0	0.9	3.5	2.6	2.2	3.3	3.5	3.6	3.0	5.6
Animal protein (g/capita/day)	***2018***	26.4	15.4	14.9	9.3	7.3	12.1	12.3	13.7	13.5	15.2	32.9
Fish/animal protein (%)	***2018***	25.0	52.2	5.7	37.6	35.3	18.5	26.5	25.3	29.1	20.0	16.9
**Contribution of aquaculture value[b]**												
Farm-gate value (million US$)	***2019***	2,862	190	64	38	833	62	242	105	4,395	4,857	259,548
Farm-gate price (US$/kg)	***2019***	1.7	3.6	3.5	4.6	2.9	3.8	2.3	2.7	2.0	2.1	3.0

Author's computation from data source [a]UN [4]; [a]World Bank [33]; [b]FishStatJ [12, 28, 31]; and [c]FAO [34]

Scenario analysis

We developed two scenarios in this study. The first scenario was business-as-usual future (*BAU*) which was characterized by a set of model parameters that reflect a continuation of past trends into the future. We had determined these trends from published sources and from the regional experts we had gathered in the consultation workshops. In our *BAU* scenario using Shared Socio Economic Pathways 2, we assumed economic development continues but is not uniform, environmental degradation continues, but at a slowing pace, and climate change presents moderate challenges to both adaptation and mitigation [32]. Under the *BAU* scenario, African economies are assumed to have a low annual income growth rate of 2.9% from 2015-2050. This *BAU* scenario replicated projection results reported in our previous study [1].

The second alternative scenario is called *high capture fisheries/aquaculture with stronger GDP growth* (*HIGH*) assumes high aquaculture growth rates being driven by substantial investment in the industry. The model was calibrated such that aquaculture output growth at 12.7% over the 2015-2030 period (a relative improvement on the 10.6% aquaculture output growth observed from the 2005 to 2015 period). This is achieved by adjusting the model's exogenous productivity growth rates of the top five aquaculture producing countries in Africa (Egypt, Nigeria, Uganda, Ghana, and Zambia) for key selected species (tilapia, mullet, and *Pangasius* and other catfish) from 2015-2050. For capture fisheries, official statistics reported that, in 2017, Africa produced 6.7 million tonnes of marine capture fisheries and 3.0 million tonnes of inland fisheries. However, reliable fisheries data, especially in Sub-Saharan Africa, is difficult to obtain. Kolding *et al*. [35] estimated substantially higher production (about 20 million tonnes) from inland fisheries based on the total freshwater resources available in Africa (e.g., lakes, rivers, reservoirs, flood plains, and swamps). Given this disparity in capture fisheries estimates, we postulate in this scenario that the potentially unaccounted capture fisheries quantities are accrued to the existing *BAU* projections. To investigate the low per capita fish consumption in Africa, this scenario also assumes an

increase in per capita incomes. Under the *HIGH* scenario we set an annual income growth rate of 4.8% per year.

Post-model estimation of employment

Employment is a key indicator for assessing socio-economic contributions of the fisheries and aquaculture sectors to food, incomes, and livelihoods. Yet, due to the informal and dispersed nature of much of the sector, quality employment data are limited for both capture fisheries and aquaculture and their value chains. To estimate direct and indirect employment in the *BAU* and *HIGH* scenarios, we reviewed national employment data from global data sets [36, 37] and national sources [38-53]. We adopted the definitions of direct and indirect employment used by the FAO [37] which suggest a full time employee is one that received 90 % of their livelihood or spends 90% of their time in that occupation; a part time employee between 30-90%, an occasional employee less than 30%, and indirect jobs are “those associated with ancillary activities such as the building of infrastructure (ponds, cages, tanks, etc.), feed and seed production, manufacturing of fish processing equipment, packaging, marketing, and distribution”. We also take indirect employment equals direct employment (full-time equivalent number of jobs) times employment multiplier.

To account for inconsistent data, we further adjusted direct and indirect employment data to better reflect our experts’ assessment of labor productivity and average employment multiplier during stakeholder consultation. We compiled the labor productivity data and used it to estimate future employment through capture fisheries. Future employment in the aquaculture sector was based on the increasing trend of labor productivity in Africa’s aquaculture sector observed over the past three years [36]. Assumptions for direct employment in aquaculture include a labor productivity/efficiency increase of 10% from 2018 to 2030, and again from 2030 to 2050 in Africa and the studied countries. This assumption

aligns with the potential technology advancement to reduce labor requirements in aquaculture and fisheries production in the future.

Post-model estimation of aquaculture investment costs

Substantial policy and investment attention has focused around the promise of aquaculture for filling the fish supply gap in Africa. For this reason, we focused explicitly on aquaculture alone to determine the size of investment needed to meet the *BAU* and *HIGH* projections of production. Aquaculture production values for the base year 2016 (except Uganda and Zambia base year in 2014) were computed using commodity prices collected from literature for each country. Production values of the base and future projections in 2030, and 2050 under the *BAU* and *HIGH* scenarios were converted to 2010 constant US dollar using the World Bank's consumer price index. The investment needed to support projected value was built on key variable input costs such as seed (i.e., the fertilized eggs, hatchlings, or fry that are spawned or caught from the wild), feed (i.e., a combination of ingredients made into a single feed to sustain and growth fish), and labor (i.e., fish farmers with full time equivalent number of direct jobs). The magnitude of costs for each scenario ascertained through literature review and validated through our expert consultation workshops. The costs of inputs were determined using farm-gate prices, average productivity, average market size, survival rate, stocking density, feed conversion ratio, average wages, input prices, and profit margins (Table 2). Future input costs were converted to constant US$ in 2010 using the consumer price index (i.e., dollar values are divided by the consumer price index of that year, and then multiplied by the index of 2010). Fixed costs such as infrastructure investment costs, and public spending for aquaculture research, development and extension, were not included in our estimation due to data limitations.

Table 2. Key parameters used for estimating the quantity and cost of key inputs in studied countries

Base year	Egypt 2016	Ghana 2016	Kenya 2016	Malawi 2016	Nigeria 2016	Tanzania 2016	Uganda 2014	Zambia 2014
Farm-gate price (US$/kg)	0.95	1.45	2.12	0.98	1.41	1.96	1.99	1.78
Feed conversion ratio (FCR)	1.12	2	1.5	1.8	1.3	1.5	1.5	1.7
Productivity (tonne/ha)	10.8	2.9	5	1.8	4	10	10	1.1
Average market size (g/fish)	300	400	400	-	800	-	-	-
Survival rate (%)	90	80	80	-	80	-	70	90
Stocking density (1000 pieces/ha)	40	-	15.6	6	6.3	30	2.5	2.8
Seed price (US$/1000 pieces)	5.6	39	62.6	6.6	57	90	70	43
Feed price (US$/kg)	0.38	0.48	0.63	0.33	0.57	0.81	0.56	0.30
Average wage (US$/year)	713	145	188	33	509	175	211	339
Profit margin (%)	34	15	23	19	28	11	37	23
References	[50, 54]	[38, 55-63]	[43, 51, 64-72]	[39, 40, 53, 73-75]	[69, 76-80]	[44, 47, 48, 81-85]	[42, 52, 86]	[45, 49, 87-89]

All values are converted to constant US$ in 2010 based on World Bank's consumer price index

Results

Scenarios

Previous projections of *BAU* scenario [1] had suggested that African capture fisheries and aquaculture production will grow at 0.2% and 1.3%, respectively, from 2015 to 2050. Despite the higher growth rate of aquaculture, capture fisheries in Africa will continue to be the main contributor to total fish production until 2050, though Egypt is a notable exception. Driven by high population growth and low GDP growth, per capita fish consumption in Africa is projected to gradually drop from 10.0 kg/year in 2015 to 7.7 kg/year in 2050 under this scenario (Table 3).

Under the *HIGH* scenario, the total capture fisheries production is projected to be 76% and 74% higher in 2030 and 2050 compared to the *BAU* scenario. This is driven by better

accounting for inland capture fisheries production, rather than substantial increases in capture fisheries production. The total aquaculture production is projected to be 350 % and 558% higher in 2030 and 2050, respectively, compared to the *BAU* projections (Table 3). With these high growth assumptions, the aquaculture production in Africa will likely surpass capture fisheries production by 2050. High GDP growth will enable purchasing power to increase per capita fish consumption from 10 kg in 2015 to 12 kg in 2030 and 14 kg in 2050 (Table 3).

Table 3. IMPACT model scenario projection of fish production and per capita fish consumption for Africa in 2015, 2030, and 2050

Region	Scenarios	Capture fisheries (million tonnes)			Aquaculture (million tonnes)			Per capita fish consumption (kg/person/year)		
		**2015**	**2030**	**2050**	**2015**	**2030**	**2050**	**2015**	**2030**	**2050**
**Africa**	*BAU*	8.7	9.0	9.2	1.8	2.4	2.9	10.0	8.5	7.7
	HIGH		15.8	16.0		11.0	18.8		12.1	14.0

Employment in fish sectors

Table 4 depicts that, overall, African capture fisheries and aquaculture sectors are estimated to sustain 20.7 million jobs (direct and indirect employment) in 2030, and generate 21.6 million jobs by 2050 under the *BAU* scenario. Direct employment in Africa's fish sector is estimated to remain relatively constant and only grow from 5.6 million in 2030 to 5.8 million in 2050 in the *BAU* scenario. In contrast, under the *HIGH* scenario, direct employment in capture fisheries and aquaculture will more than double, to 12.2 million, by 2050, where for every person directly employed in the sector, 2.6 people will be indirectly employed. By 2050, capture fisheries and aquaculture sectors sustain 58[illegible]0 million jobs. Under the *BAU* and *HIGH* scenarios, the total direct and indirect employment for the fish sector will represent 0.9% and 2.4%, respectively, of the total projected 2.4 billion African in 2050.

For direct employment in African aquaculture, even with increasing average labor productivity from 5.8 tonnes/worker in 2030 to 6.3 tonnes/worker in 2050, fish farmers are projected to increase to 0.3 million under *BAU* and sharply increase to 1.1 million under *HIGH* by 2050 due to the 71% higher in aquaculture production. Among the eight studied countries, Egypt has the highest labor productivity of 13-15 tonnes/worker, followed by Uganda, Nigeria, Ghana, and Zambia. Conversely, Malawi, Tanzania, and Kenya have relatively lower labor efficiency with less than one tonne/worker (Table 5).

Employment generated by capture fisheries contributes to over 90% of the total jobs in the African fish sector under *BAU*. This is mainly due to higher capture fisheries output but lower labor productivity as compared to aquaculture. Among the studied countries, Egypt again has the highest labor efficiency in capture fisheries of 9.3 tonnes/worker (Table 5 and 6). Egypt is the only country that has a higher proportion of jobs generated by aquaculture than capture fisheries in the *HIGH* scenario (Fig 1). Nigeria is among the studied countries that generates the highest total employment in the fish sector, particularly in the capture fisheries sector. Only two countries—Nigeria and Zambia—have average employment multipliers less than one, resulting in the proportion of indirect employment as less than half of the total employment in the fish sector (Fig 2).

>>Insert Figures 1 and 2 here>>

Figure 1. Direct employment of capture fisheries and aquaculture under *BAU* scenario in 2030 (A), *BAU* scenario in 2050 (B), *HIGH* scenario in 2030 (C), and *HIGH* scenario in 2050 (D).

Figure 2. Direct and indirect employment of fish sector under *BAU* scenario in 2030 (A), *BAU* scenario in 2050 (B), *HIGH* scenario in 2030 (C), and *HIGH* scenario in 2050 (D).

Table 4. Estimated direct and indirect employment of Africa's fish food system for *BAU* and *HIGH* scenarios in 2030 and 2050

Country	Scenarios	Fish production (thousand tonnes)		Direct labor productivity (tonnes/worker)		Direct employment (thousand)		Average employment multiplier		Indirect employment (thousand)		Total direct and indirect employment (thousand)	
		2030	2050	2030	2050	2030	2050	2030	2050	2030	2050	2030	2050
**Africa**	*BAU*	11,439	12,064	2.0	2.1	5,630	5,774	2.6	2.7	15,035	15,855	20,665	21,629
	HIGH	26,784	34,816	2.4	2.8	11,049	12,230	3.2	3.7	35,202	45,758	46,251	57,988
**Egypt**	*BAU*	1,924	2,169	12.4	13.4	156	161	1.8	2.0	288	324	443	485
	HIGH	4,977	7,632	12.8	14.1	389	540	1.7	2.0	680	1,078	1,069	1,617
**Ghana**	*BAU*	369	389	1.4	1.4	271	276	2.1	2.2	565	597	835	872
	HIGH	1,147	1,722	1.7	2.2	660	790	2.7	3.3	1,757	2,639	2,417	3,429
**Kenya**	*BAU*	209	213	1.9	1.9	111	113	2.0	2.0	217	222	328	335
	HIGH	618	630	2.7	2.6	228	242	2.8	2.7	644	656	872	897
**Malawi**	*BAU*	133	136	1.0	1.0	135	137	4.0	4.0	534	545	669	682
	HIGH	397	404	1.0	1.0	400	406	4.0	4.0	1,595	1,622	1,995	2,028
**Nigeria**	*BAU*	1,441	1,638	1.3	1.3	1,137	1,266	0.9	0.9	1,005	1,142	2,142	2,409
	HIGH	2,072	2,396	1.2	1.3	1,749	1,903	0.8	0.9	1,445	1,670	3,194	3,573
**Tanzania**	*BAU*	341	341	1.8	1.8	192	192	1.3	1.3	247	247	439	439
	HIGH	1,087	1,088	1.8	1.8	602	604	1.3	1.3	786	787	1,409	1,416
**Uganda**	*BAU*	639	669	3.6	3.6	179	183	3.9	4.0	693	726	872	909
	HIGH	2,070	2,151	3.4	3.4	616	632	3.6	3.7	2,245	2,333	2,861	2,965
**Zambia**	*BAU*	115	124	1.3	1.3	92	96	0.5	0.5	49	52	141	148
	HIGH	418	474	1.2	1.3	347	377	0.5	0.5	177	200	524	578

Table 5. Estimated direct employment of Africa's aquaculture sector for *BAU* and *HIGH* scenarios in 2030 and 2050

Country	Scenarios	Aquaculture production (thousand tonnes)		Labor productivity (tonnes/worker)	Labor productivity (tonnes/worker)	Direct employment	
		2030	2050	2030	2050	2030	2050
**Africa**	*BAU*	2,439	2,864	5.8	6.3	424,368	452,866
	HIGH	10,984	18,816			1,910,711	2,975,598
**Egypt**	*BAU*	1,594	1,843	13.3	14.6	120,303	126,449
	HIGH	4,550	7,210			343,318	494,569
**Ghana**	*BAU*	85	102	3.9	4.2	21,929	24,067
	HIGH	558	1,122			144,856	264,663
**Kenya**	*BAU*	33	38	0.6	0.6	60,080	62,722
	HIGH	33	45			60,063	73,717
**Malawi**	*BAU*	8	9	0.8	0.9	9,328	10,292
	HIGH	8	11			9,328	12,124
**Nigeria**	*BAU*	445	526	4.5	5.0	98,846	106,190
	HIGH	500	707			111,188	142,952
**Tanzania**	*BAU*	7	8	0.8	0.9	9,010	9,315
	HIGH	7	10			9,005	10,822
**Uganda**	*BAU*	134	154	5.4	5.9	25,071	26,207
	HIGH	136	184			25,468	31,305
**Zambia**	*BAU*	29	34	1.8	1.9	16,379	17,610
	HIGH	61	103			34,658	53,325

Table 6. Estimated direct employment of Africa's capture fisheries sector for *BAU* and *HIGH* scenarios in 2030 and 2050

Country	Scenarios	Capture fisheries production (thousand tonnes)		Labor productivity (tonnes/worker)	Direct employment	
		2030	2050		2030	2050
**Africa**	*BAU*	9,000	9,200	1.7	5,205,551	5,321,230
	HIGH	15,800	16,000		9,138,634	9,254,313
**Egypt**	*BAU*	330	326	9.3	35,328	34,869
	HIGH	427	422		45,690	45,231
**Ghana**	*BAU*	284	287	1.1	248,730	251,645
	HIGH	588	600		515,164	525,665
**Kenya**	*BAU*	176	175	3.5	50,456	50,415
	HIGH	585	585		168,211	168,170
**Malawi**	*BAU*	125	126	1.0	125,534	126,717
	HIGH	389	393		385,213	393,786
**Nigeria**	*BAU*	996	1,113	1.0	1,038,293	1,159,992
	HIGH	1,572	1,688		1,638,155	1,759,854
**Tanzania**	*BAU*	334	333	1.8	183,254	182,948
	HIGH	1,079	1,079		592,958	592,680
**Uganda**	*BAU*	505	515	3.3	154,162	157,281
	HIGH	1,934	1,967		590,683	600,731
**Zambia**	*BAU*	86	90	1.1	75,607	78,438
	HIGH	357	370		312,323	324,017

Aquaculture production values and investment costs

Under the *BAU* scenario, Africa's aquaculture production is projected to reach 2.4 million tonnes, valued at US$ 2.8 billion, and 2.9 million tonnes, valued at US$ 3.3 billion in 2030 and 2050, respectively. Farm-gate investment costs for three key variable inputs of feed, labor, and fish seed to realize aquaculture production in 2030 and 2050 are shown in Table 7. The investment costs are projected to increase to US$ 1.6 billion and US$ 1.8 billion in 2030 and 2050, respectively, to achieve the projected aquaculture outputs in those years. Of the three key variable costs estimated, feed costs account for 81% to 84%, labor costs range from 10% to 12%, and fish seed costs a little over 6% (Fig 3). The investment cost structure is likely to remain the same, unless there are technological innovations in the fish feed and seed sectors, resulting in a substantial decrease in feed costs.

>>Insert Figure 3 here>>

Figure 3. Cost structure of key input production costs of aquaculture in Africa and studied countries

Under the *HIGH* scenarios, aquaculture production values in Africa are projected to reach US$ 11.9 billion in 2030 and US$ 20.4 billion in 2050 (Table 7). To maintain the aquaculture growth rate as projected in the *HIGH* scenario, investment costs in the three key variable costs of feed, labor, and seed need to increase to US$ 6.8 billion by 2030 and US$ 11.6 billion by 2050. These key investment costs will be invested by producers, including private aquaculture enterprises and farmers at different production scales. Similar to the *BAU* scenario, feed cost is the main component, accounting for more than 80% (Fig 3). Investing in aquaculture feed is critical to achieving the aquaculture production outputs by 2030 and 2050 projected in the *HIGH* scenario.

Our post-model estimation (Table 7) suggests that uneven distribution of future aquaculture production values and required investment costs will remain under both the *BAU* and *HIGH* scenarios. Under the *BAU*, the eight countries included in this study are projected to account for 96% of Africa's aquaculture production values in 2030 and only slightly reduce to 95% by 2050. A similar pattern is observed for investment costs of the key variable inputs required to achieve aquaculture projection output levels. The top four African aquaculture producers–namely Egypt, Nigeria, Uganda, and Ghana–account for 90% of the production values while the group of Kenya, Zambia, Tanzania, and Malawi was projected to account for 5% throughout 2030 and 2050.

Table 7. Annual output value and key inputs costs of Africa's aquaculture for *BAU* and *HIGH* scenarios in 2030 and 2050

Country	Scenarios	Aquaculture production values (million US$)		2030 farm-gate costs (million US$)				2050 farm-gate costs (million US$)			
		2030	2050	Feed	Labor	Seed	Total	Feed	Labor	Seed	Total
**Africa**	*BAU*	2,799.4	3,290.0	1,313.6	170.6	101.5	**1,585.7**	1,545.9	182.4	120.1	**1,848.4**
	HIGH	11,862.8	20,373.9	5,670.3	661.7	421.5	**6,753.5**	9,859.1	1,010.8	751.7	**11,621.6**
**Egypt**	*BAU*	1,518.8	1,756.1	672.9	85.8	33.2	**791.9**	778.0	90.2	38.4	**906.6**
	HIGH	4,334.4	6,868.3	1,920.2	244.8	94.8	**2,259.8**	3,042.8	352.6	150.3	**3,545.7**
**Ghana**	*BAU*	122.7	148.1	81.8	3.2	10.2	**95.2**	98.7	3.5	12.3	**114.5**
	HIGH	810.2	1,628.4	540.1	21.0	67.5	**628.6**	1,085.6	38.4	135.7	**1,259.7**
**Kenya**	*BAU*	70.0	80.4	31.0	11.3	6.5	**48.8**	35.7	11.8	7.4	**54.9**
	HIGH	70.0	94.5	31.0	11.3	6.5	**48.8**	41.9	13.9	8.7	**64.5**
**Malawi**	*BAU*	7.7	9.3	4.6	0.3	0.2	**5.1**	5.6	0.3	0.2	**6.1**
	HIGH	7.7	11.0	4.6	0.3	0.2	**5.1**	6.6	0.4	0.2	**7.2**
**Nigeria**	*BAU*	627.6	741.7	330.2	50.3	39.5	**420.0**	390.2	54.0	46.6	**490.8**
	HIGH	706.0	998.4	371.4	56.6	44.4	**472.4**	525.3	72.7	62.8	**660.8**
**Tanzania**	*BAU*	14.5	16.5	8.9	1.6	2.0	**12.5**	10.2	1.6	2.3	**14.1**
	HIGH	14.5	19.2	8.9	1.6	2.0	**12.5**	11.8	1.9	2.6	**16.3**
**Uganda**	*BAU*	266.7	306.6	113.4	5.3	2.4	**121.1**	130.4	5.5	2.7	**138.6**
	HIGH	270.9	366.3	115.2	5.4	2.4	**123.0**	155.7	6.6	3.2	**165.5**
**Zambia**	*BAU*	51.5	60.9	14.5	5.6	3.2	**23.3**	17.1	6.0	3.8	**26.9**
	HIGH	109.0	184.3	30.7	11.8	6.9	**49.4**	51.9	18.1	11.6	**81.6**

All value costs are in millions of constant 2010 US$

Discussion

One of Africa's biggest development challenges is to meet the nutrition needs, within sustainable limits of 2.4 billion women, men and children by 2050 [90]. Experiences in Asia and other regions show that aquatic foods, capture fisheries, and aquaculture systems [91] offer important nutritional and sustainability values, in some cases outperforming nutritional qualities of dietary supplements [92] and a relatively lower environmental footprint than animal-source foods [93, 94]. However, the role of fisheries, aquaculture and aquatic foods in the transformation of food systems has remained relatively overlooked due to the lack of scientific data, metrics, and evidence to inform donors, governments, and private investors in decision making and investment planning [95]. Our objective in this paper is to employ a rigorous partial equilibrium economic modeling tool, the IMPACT fish model, to generate future fish supply-demand projections in Africa to 2050 under the *BAU* and *HIGH* scenarios. Fish supply projections are then used to extrapolate future direct and indirect employment and investment costs needed to achieve the projected output levels.

There is a global concern that current food systems are ill-equipped to deliver nutritious food, a challenge that will be exacerbated as demands of burgeoning populations and wealth will outpace supplies. In practice, limited supplies of quality food will affect different people to different degrees based on their economic status, geography, and gender, with women of reproductive age and children under the age of five being most vulnerable to nutrient deficiencies [96]. Our *BAU* scenario projection shows that per capita fish consumption in Africa will gradually drop from 10.0 kg/person/year (about half the global and Asian fish intake) in 2015 to 7.7 kg/person/year in 2050. The drop in fish consumption we see in our *BAU* scenario is a result of population growth outpacing growth in the fish sector. Decreasing per capita fish consumption is also the outcome of modest GDP growth. Lower economic growth will constrain governments' and private sector's ability to invest in supply infrastructure, technology, and management systems that might otherwise boost supplies. In the optimistic

HIGH scenario, with GDP growth at 4.8% per year to 2050, per capita fish consumption is projected to increase from 10.0 kg/year in 2015 to 14.0 kg/year in 2050. Investments in sustainable fisheries management and aquaculture will boost total domestic production to 34.8 million tonnes in 2050, of which the share of capture fisheries in total fisheries production in Africa will decline from 82.7% in 2015 to 46.0% in 2050. These results show that there is potential to sustainably increase capture fisheries and expand aquaculture to meet the growing demand for fish in Africa. This needs a sound enabling macro-environment, particularly moderate to high economic growth to stimulate fish demand increase and sustained investment from farmers, investors and governments to transform Africa's capture fisheries and aquaculture into sustainable, productive, nutrition-sensitive and inclusive aquatic food systems.

Youth employment, and the future employment of current youth, is a growing opportunity and concern globally, and aquaculture and fisheries offer possible but evolving opportunities. With rapid population growth and a young population (60% of the African population below the age of 25), it is expected that 11 million young people will enter the job market in Sub-Saharan Africa every year, while only about 3 million new jobs are created annually on the continent [97-99]. Both capture fisheries and aquaculture are important sources of employment in Africa, particularly for smallholders and value chain actors in rural areas [97]. Creating jobs in rural areas at a large-scale is critical to address these unemployment issues and income generation in Africa. Our study results show that under the *BAU* scenario, with slow aquaculture growth and almost stagnant capture fisheries, Africa's fish sector is projected to provide 22 million direct and indirect jobs by 2050. However, with the *HIGH* scenario, 58 million people will be directly and indirectly employed in fisheries and aquaculture sectors, representing 2.4% of the total projected population in Africa in 2050. The projection results indicate that growth in Africa's fish sector will create considerable employment and has the potential to generate significant income growth and facilitate inclusive value chain development to address development barriers faced by Africa. About 60 million

people (14% of whom are women) were engaged in the primary sector to produce 179 million tonnes of fish globally in 2018 [100], implying a global average labor productivity of 3.0 tonnes/worker. Our projection estimated that the overall African labor productivity of direct employment in both capture fisheries and aquaculture is 2.0 tonnes/worker, slightly lower than the world average. Furthermore, employment in the fish sector in Africa will continue to be dominated by small-scale fisheries, with lower labor productivity compared to aquaculture (1.7 tonnes/worker vs. 6.3 tonnes/worker). This result highlights the importance of sustainable capture fisheries management to generate employment opportunities and provide income for the portion of the African population depending on artisanal fishing. In order to achieve the desired sustainability transformation, public policy leadership and private sector technological innovation will be required [101].

Our projection results show that strong aquaculture growth has a high potential to generate income and jobs for rural communities in Africa. Under the *HIGH* scenario, aquaculture production in Africa is projected to reach 18.8 million tonnes, generating a revenue of US$ 20.4 billion in 2050. Projected farm-gate investment costs of three key aquaculture inputs (feed, labor, and fish seed) are at US$ 11.6 billion in 2050. It is essential to highlight that these investments can be mobilized from farmers, private sector investors and enterprises, suggesting dynamic opportunities for market-led aquaculture business development. Given that feed accounts for a major share of aquaculture production cost, this suggests that there will be bright prospects for investing in the aquaculture feed industry in Africa. It will be essential to have more supportive policies and regulations to serve as an entry point for the private sector on more inclusive ways to engage smallholders in the fish value chains.

This study provides useful insights on how aquatic foods, fisheries and aquaculture systems in Africa might evolve into the future under complex and dynamic interactions of structural changes, technological progress, income growth, and urbanization in a climate crisis. The study findings also allow drawing policy implications of different impact pathways, drivers

and interventions to enhance aquatic food systems' contributions to sustainable development goals in Africa. Notwithstanding these contributions, our study has several limitations due to data gaps. First, in aquaculture investment cost extrapolation, we do not estimate the required investments for farm or value chain infrastructure. Second, we are unable to project investment costs needed to sustain capture fisheries, which mostly come from public funding and philanthropic investment. Future follow-up studies should investigate aquaculture infrastructure cost requirements and investment costs for capture fisheries management in Africa. Third, our extrapolation of outcomes focuses only on employment opportunities. Enabling women to fully engage in and benefit from small-scale fisheries and aquaculture can boost production, reduce poverty, and improve food and nutrition security in Africa [102]. Further research is needed to extend the post-model analysis to examine implications on other outcome areas such as gender equity, nutrition and environmental sustainability associated with different future projected trends. Effective and efficient use of data collection tools for gender and youth assessment needs to be embedded in a future inclusive development process. Fourth, our estimation of investment costs does not include public investment in infrastructure, human capital and research capacity needed to create macro and micro enabling environment for aquaculture and fisheries sector performance. Finally, aquatic foods are relatively new in the realm of foresight modeling tools compared to crops and livestock, and further advancement of fish foresight modeling tools is essential to improve the quality of modeling projections and incorporating these outcomes in future analyses.

Conclusions

Our current food systems face severe challenges in achieving equitable access to healthy, nutritious food, maintaining environmental sustainability, and building resilience to shocks. Fish and aquatic foods offer significant potential in the transformation of food systems toward healthy and sustainable diets, sustaining livelihoods, and generating income. The fish sectors

are important for employment creation in Africa, yet, their role has been overlooked, resulting in insufficient investment to support the sector growth and sustainable system transformation to meet the increasing demand for fish. This study provides insights into future fish supply and demand projections in Africa under the *BAU* and *HIGH* scenarios and provides first estimates of employment generated and the necessary input cost investments required to secure projected fish supplies in 2030 and 2050. The study suffers limitations that should be addressed in the future. Nonetheless, this is a key and first analysis to look at macro-level employment and investment scenarios of fish sectors in Africa.

Acknowledgments

The authors would like to thank the contribution of a wide range of African government, academia, non-profit organizations who provide valuable information and feedback to this research work. In particular, Dr. Richard Abila from IFAD; Dr. Alexander Shula Kefi from Department of Fisheries Zambia; Dr. Diaa Al-Kenawy from WorldFish Egypt office, Dr. Bernadette Tosan Fregene from University of Ibadan, Nigeria; Dr. Emmanuel Nii Abbey from University of Ghana, Dr. Mafaniso Hara from University of Western Cape, Dr. Amon Paul Shoko from Tanzania Fisheries Research Institute, and Dr. Anthony Dadu from Department of Aquaculture, Ministry of Agriculture, Livestock and Fisheries, Tanzania. We would like to also thank Dr. Michael Phillips for his support to this research implementation process, and Dr Junning Cai and Jennifer Gee from FAO for sharing fish sector employment datasets and information.

Author Contributions

Conceptualization: Nhuong Tran, Chin Yee Chan, Philippa J Cohen

Data Curation: Kai Ching Cheong, Chin Yee Chan, Nhuong Tran

Formal Analysis: Chin Yee Chan, Kai Ching Cheong, Nhuong Tran

Funding Acquisition: Nhuong Tran, Chin Yee Chan

Investigation: Chin Yee Chan, Kai Ching Cheong, Nhuong Tran

Methodology: Chin Yee Chan, Nhuong Tran, Kai Ching Cheong, Timothy B Sulser

Project Administration: Chin Yee Chan, Nhuong Tran, Kai Ching Cheong, Ahmed Mohamed Nasr Allah

Resources: Chin Yee Chan, Nhuong Tran, Kai Ching Cheong

Software: Chin Yee Chan, Nhuong Tran, Kai Ching Cheong

Supervision: Nhuong Tran

Validation: Chin Yee Chan, Nhuong Tran, Kai Ching Cheong, Ahmed Mohamed Nasr Allah, Timothy B Sulser

Visualization: Chin Yee Chan, Nhuong Tran, Kai Ching Cheong

Writing – Original Draft Preparation: Chin Yee Chan, Nhuong Tran

Writing – Review & Editing: Nhuong Tran, Chin Yee Chan, Philippa J Cohen, Timothy B Sulser, Kai Ching Cheong, Keith Wiebe, Ahmed Mohamed Nasr-Allah

References

1. Chan CY, Tran N, Pethiyagoda S, Crissman CC, Sulser TB, Phillips MJ. Prospects and challenges of fish for food security in Africa. Global Food Security. 2019;20:17-25. https://doi.org/10.1016/j.gfs.2018.12.002

2. Springmann M, Clark M, Mason-D'Croz D, Wiebe K, Bodirsky BL, Lassaletta L, et al. Options for keeping the food system within environmental limits. Nature. 2018. https://doi.org/10.1038/s41586-018-0594-0

3. Troell M, Naylor RL, Metian M, Beveridge M, Tyedmers PH, Folke C, et al. Does aquaculture add resilience to the global food system? Proc Natl Acad Sci U S A. 2014;111(37):13257-63. https://doi.org/10.1073/pnas.1404067111

4. UN. Department of Economic and Social Affairs, Population Division. World Population Prospects 2019. 2019. Available from: https://population.un.org/wpp

5. African Development Bank. Africa in Fifty Year's Time: The road towards inclusive growth. Tunis, Tunisia. 2011:76. Available from: https://www.afdb.org/fileadmin/uploads/afdb/Documents/Publications/Africa%20in%2050%20Years%20Time.pdf

6. Obiero K, Meulenbroek P, Drexler S, Dagne A, Akoll P, Odong R, et al. The Contribution of Fish to Food and Nutrition Security in Eastern Africa: Emerging Trends and Future Outlooks. Sustainability. 2019;11(6). https://doi.org/10.3390/su11061636

7. O'Meara L, Cohen PJ, Simmance F, Marinda P, Nagoli J, Teoh SJ, et al. Inland fisheries critical for the diet quality of young children in sub-Saharan Africa. Global Food Security. 2021;28. https://doi.org/10.1016/j.gfs.2020.100483

8. Tran N, Chu L, Chan CY, Genschick S, Phillips MJ, Kefi AS. Fish supply and demand for food security in Sub-Saharan Africa: An analysis of the Zambian fish sector. Marine Policy. 2019;99:343-50. https://doi.org/10.1016/j.marpol.2018.11.009

9. Willett W, Rockström J, Loken B, Springmann M, Lang T, Vermeulen S, et al. Food in the Anthropocene: the EAT-Lancet Commission on healthy diets from sustainable food systems. The Lancet. 2019;393(10170):447-92. https://doi.org/10.1016/s0140-6736(18)31788-4

10. HLPE. Sustainable fisheries and aquaculture for food security and nutrition. A report by the high level panel of experts on food security and nutrition of the committee on world food security Rome: FAO. 2014. Available from: http://www.fao.org/3/i3844e/i3844e.pdf

11. Funge-Smith SJ. Review of the state of world fishery resources: inland fisheries. FAO Fisheries and Aquaculture Circular No. C942 Rev.3, Rome. 397 pp. 2018. Available from: http://www.fao.org/3/CA0388EN/ca0388en.pdf

12. FAO. Fishery and Aquaculture Statistics. Global aquaculture production 1950-2019 (FishstatJ). In: FAO Fisheries and Aquaculture Department [online]. Rome. 2021. Available from: www.fao.org/fishery/statistics/software/fishstatj/en

13. Naylor RL. Oil crops, aquaculture, and the rising role of demand: A fresh perspective on food security. Global food security. 2016;11:17-25. https://dx.doi.org/10.1016/j.gfs.2016.05.001

14. Thurstan RH, Roberts CM. The past and future of fish consumption: Can supplies meet healthy eating recommendations? Mar Pollut Bull. 2014;89(1-2):5-11. https://doi.org/10.1016/j.marpolbul.2014.09.016

15. Victora CG, Christian P, Vidaletti LP, Gatica-Domínguez G, Menon P, Black RE. Revisiting maternal and child undernutrition in low-income and middle-income countries:

variable progress towards an unfinished agenda. The Lancet. 2021;397(10282):1388-99. https://doi.org/10.1016/s0140-6736(21)00394-9

16. Béné C, Lawton R, Allison EH. “Trade Matters in the Fight Against Poverty”: Narratives, Perceptions, and (Lack of) Evidence in the Case of Fish Trade in Africa. World Development. 2010;38(7):933-54. https://doi.org/10.1016/j.worlddev.2009.12.010

17. Chan CY, Tran N, Dao CD, Sulser TB, Phillips MJ, Batka M, et al. *Fish to 2050* in the ASEAN region. Penang, Malaysia: WorldFish and Washington DC, USA: International Food Policy Research Institute (IFPRI) Working Paper. 2017;2017-01. Available from: http://pubs.iclarm.net/resource_centre/2017-01.pdf

18. Hope KR. Climate change and poverty in Africa. International Journal of Sustainable Development & World Ecology. 2009;16(6):451-61. https://doi.org/10.1080/13504500903354424

19. Barange M, Cheung WWL, Merino G, Perry RI. Modelling the potential impacts of climate change and human activities on the sustainability of marine resources. Current Opinion in Environmental Sustainability. 2010;2(5-6):326-33. https://doi.org/10.1016/j.cosust.2010.10.002

20. Cheung WWL, Lam VWY, Sarmiento JL, Kearney K, Watson REG, Zeller D, et al. Large-scale redistribution of maximum fisheries catch potential in the global ocean under climate change. Global Change Biology. 2010;16(1):24-35. https://doi.org/10.1111/j.1365-2486.2009.01995.x

21. Lam VWY, Cheung WWL, Swartz W, Sumaila UR. Climate change impacts on fisheries in West Africa: implications for economic, food and nutritional security. African Journal of Marine Science. 2012;34(1):103-17. https://doi.org/10.2989/1814232x.2012.673294

22. Hollowed AB, Barange M, Beamish RJ, Brander K, Cochrane K, Drinkwater K, et al. Projected impacts of climate change on marine fish and fisheries. ICES Journal of Marine Science. 2013;70(5):1023-37. https://doi.org/10.1093/icesjms/fst081

23. IPCC. Climate change 2007: Impacts, adaptations and vulnerability. In M. L. Parry, O. F. Canzian, J. P. Palutikof, P. J. van der Linden, J. Paul & C. Hanson (Eds.), Contribution of working group II to the 4th assessment report of the Intergovernmental Panel on Climate Change (IPCC). Cambridge: Cambridge University Press. 2007.

24. IPCC. Climate change: impacts, adaptation and vulnerability. In Working group II contribution to the intergovernmental panel on climate change. Summary for policy makers. IPCC Secretariat, Geneva, Switzerland. 2007. Available from: https://www.ipcc.ch/working-group/wg2/

25. Belhabib D, Cheung WWL, Kroodsma D, Lam VWY, Underwood PJ, Virdin J. Catching industrial fishing incursions into inshore waters of Africa from space. Fish and Fisheries. 2019;21(2):379-92. https://doi.org/10.1111/faf.12436

26. Belton B, Rosen L, Middleton L, Ghazali S, Mamun A-A, Shieh J, et al. COVID-19 impacts and adaptations in Asia and Africa's aquatic food value chains. Marine Policy. 2021;129. https://doi.org/10.1016/j.marpol.2021.104523

27. NEPAD. Planning and Coordinating Agency, African Union Inter-African Bureau for Animal Resources. The Pan-African Fisheries and Aquaculture Policy Framework and Reform Strategy: Promoting Inter-Regional Fish Trade. NPCA, AU-IBAR, Midrand, South Africa. 2016.

28. FAO. Fishery and Aquaculture Statistics. Food Balance Sheets 1961-2017 (FishstatJ). In: FAO Fisheries and Aquaculture Department [online]. Rome. 2021. Available from: www.fao.org/fishery/statistics/software/fishstatj/en

29. Delgado CL, Wada N, Rosegrant MW, Meijer S, Ahmed M. Fish to 2020: Supply and Demand in Changing Global Markets: WorldFish Center Technical Report 62. Washington, DC: International Food Policy Research Institute; 2003. Available from: http://pubs.iclarm.net/resource_centre/WF_356.pdf

30. World Bank. Fish to 2030: Prospect for Fisheries and Aquaculture. Washington, DC: World Bank. 2013. Available from: http://documents1.worldbank.org/curated/en/458631468152376668/pdf/831770WP0P11260ES003000Fish0to02030.pdf

31. FAO. Fishery and Aquaculture Statistics. Global capture production 1950-2019 (FishstatJ). In: FAO Fisheries and Aquaculture Department [online]. Rome. 2021. Available from: www.fao.org/fishery/statistics/software/fishstatj/en

32. IFPRI. Repository for IFPRI's IMPACT model. 2020. Available from: https://github.com/IFPRI/IMPACT

33. World Bank. World Bank Open Data. 2021. Available from: https://data.worldbank.org

34. FAO. FAOSTAT statistics database. Food balance sheets. 2021. Available from: http://www.fao.org/faostat/en/#data/FBS

35. Kolding J, Zwieten PAMv, Mosepele K. "Where there is water there is fish". Small-scale inland fisheries in Africa: dynamics and importance. In: Tvedt T, Oestigaard T, editors. A History of Water Vol 3 Water and Food: From hunter-gatherers to global production in Africa. London: I.B. Tauris; 2015.

36. FAO. The State of World Fisheries and Aquaculture 2018 - Meeting the sustainable development goals. Rome. 2018. Available from: http://www.fao.org/3/i9540en/i9540en.pdf

37. Valderrama D, Hishamunda N, Zhou X. Estimating Employment in World Aquaculture. FAO Aquaculture Newsletter No 45. 2011:24-5. Available from: http://www.fao.org/3/al363e/al363e12.pdf

38. Aheto DW, Acheampong E, Odoi JO. Are small-scale freshwater aquaculture farms in coastal areas of Ghana economically profitable? Aquaculture International. 2019;27(3):785-805. https://doi.org/10.1007/s10499-019-00363-9

39. Anker R, Anker M. Living Wage for rural Malawi with Focus on Tea Growing area of Southern Malawi. Fairtrade International, Sustainable Agriculture Network/Rainforest Alliance and UTZ Certified, 70. 2014.

40. Dey MM, Paraguas FJ, Kambewa P, Pemsl DE. The impact of integrated aquaculture-agriculture on small-scale farms in Southern Malawi. Agricultural Economics. 2010;41(1):67-79. https://doi.org/10.1111/j.1574-0862.2009.00426.x

41. Hasan MR, Hecht T, De Silva SS, Tacon AGJ. Study and analysis of feeds and fertilizers for sustainable aquaculture development. FAO Fisheries Technical Paper No 497 Rome, FAO 2007. 2007:510p. Available from: http://www.fao.org/3/a1444e/a1444e00.htm

42. Hyuha TS, Egna H, Ekere W. Social and economic performance of tilapia farming in Uganda. In J. Cai, K. K. Quagrainie & N. Hishamunda, eds. Social and economic performance of tilapia farming in Africa, pp.127–144. FAO Fisheries and Aquaculture Circular No 1130 Rome, Italy. 2017. Available from: http://www.fao.org/3/a-i7258e.pdf

43. Jacobi N. Examining the Potential of Fish Farming to Improve the Livelihoods of Farmers in the Lake Victoria Region, Kenya - Assessing Impacts of Governmental Support. Faculty of Business and Science University of Akureyri, University Centre of the Westfjords, Master of Resource Management: Coastal and Marine Management Ísafjörður, May 2013. 2013. Available from: https://aquadocs.org/bitstream/handle/1834/6854/ktf0232.pdf?sequence=1

44. Kaliba AR, Osewe KO, Senkondo EM, Mnembuka BV, Quagrainie KK. Economic Analysis of Nile Tilapia (Oreochromis niloticus) Production in Tanzania. Journal of the World Aquaculture Society. 2006;37(4):464-73. https://doi.org/10.1111/j.1749-7345.2006.00059.x

45. Kaminski AM, Genschick S, Kefi AS, Kruijssen F. Commercialization and upgrading in the aquaculture value chain in Zambia. Aquaculture. 2018;493:355-64. https://doi.org/10.1016/j.aquaculture.2017.12.010

46. Kofi F, Nunoo E, Asamoah EK, Osei-asare YB. Economics of aquaculture production: a case study of pond and pen culture in southern Ghana. Aquaculture Research. 2014;45:675-88. https://doi.org/10.1111/are.12003

47. Kyelu A. Analysis of socio-economic and environmental effects of urban fish farming in Dar es Salaam, Tanzania. Sokoine University of Agriculture. 2016. Available from: http://www.suaire.sua.ac.tz/handle/123456789/1428

48. Masike S, Liwenga E. Cost Benefit Analysis of the Community Livelihoods Improvement Initiatives in Tanzania. Pro-poor Economic Growth and Environmentally Sustainable Development Project. 2017. Available from: https://pea4sdgs.org/sites/default/files/2020-09/Cost%20Benefit%20Analysis%20of%20the%20Community%20Livelihoods%20Improvement%20Initiatives%20in%20Tanzania_2.pdf

49. Mwango J, Kefi AS, Manda EC, Chijoka M, Chimba N. Status of aquaculture in the Copperbelt and North-western provinces of Zambia. Republic of Zambia Ministry of Fisheries and Livestock. 2016:1-68. Available from: https://www.academia.edu/30644672/STATUS_OF_AQUACULTURE_IN_THE_COPPERBELT_AND_NORTH_WESTERN_PROVINCES_OF_ZAMBIA

50. Nasr-Allah A, Macfadyen G, Dickson M, Al–Kenawy D, Fathi M, El-Naggar G. Value Chain Analysis of the Egyptian Aquaculture Sector. IIFET 2012 Tanzania Proceedings. 2012. https://doi.org/10.13140/2.1.3709.2808

51. Nyandat B, Owiti GO. Aquaculture needs assessment mission report. Report/Rapport: SF-FAO/2013/24. September/Septembre 2013. FAO-SmartFish Programme of the Indian Ocean Commission, Ebene, Mauritius. 2013. Available from: http://www.fao.org/3/az041e/az041e.pdf

52. Safina N, Gertrude A, Lawrance O, Ronald W, Alphonse C, Samuel O, et al. Profitability and Viability Analysis of Aquaculture Production in Central Uganda: A Case of Urban and Peri-Urban Areas. Asian Journal of Agricultural Extension, Economics & Sociology. 2018;22(4):1-11. https://doi.org/10.9734/ajaees/2018/37721

53. Tran N, Crissman C, Chijere A, Hong MC, Teoh SJ, Valdivia RO. Ex-ante assessment of integrated aquaculture-agriculture adoption and impact in Southern Malawi. Working paper: AAS-2013-03, CGIAR. 2013. Available from: http://pubs.iclarm.net/resource_centre/WF_3540.pdf

54. Kleih U, Linton J, Marr A, Mactaggart M, Naziri D, Orchard JE. Financial services for small and medium-scale aquaculture and fisheries producers. Marine Policy. 2013;37:106-14. https://doi.org/10.1016/j.marpol.2012.04.006

55. Amankwah A, Quagrainie KK. Aquaculture feed technology adoption and smallholder household welfare in Ghana. Journal of the World Aquaculture Society. 2018:1-15. https://doi.org/10.1111/jwas.12544

56. Amenyogbe E, Chen G, Wang Z, Lin M, Lu X, Atujona D, et al. A Review of Ghana's Aquaculture Industry. Journal of Aquaculture Research & Development. 2018;09(08). https://doi.org/10.4172/2155-9546.1000545

57. Anane-Taabeah G, Quagrainie K, Amisah S. Assessment of farmed tilapia value chain in Ghana. Aquaculture International. 2015;24(4):903-19. https://doi.org/10.1007/s10499-015-9960-1

58. FAO. Fishery and Aquaculture Country Profiles - The Republic of Ghana. FAO Fisheries & Aquaculture. 2016. Available from: http://www.fao.org/fi/oldsite/FCP/en/GHA/profile.htm

59. Karikari AY. Assessment of Environmental Impacts of Cage Aquaculture on Lake Volta of Ghana. Kwame Nkrumah University of Science and Technology. 2016. Available from: http://129.122.16.11/bitstream/123456789/9976/1/FINAL%20THESIS.pdf

60. Kassam L, Dorward A. A comparative assessment of the poverty impacts of pond and cage aquaculture in Ghana. Aquaculture. 2017;470:110-22. https://doi.org/10.1016/j.aquaculture.2016.12.017

61. Mensah ETD, Dankwa HR, Torben LL, Asmah R, Campion BB, Edziyie R. Effects of seasonal and environmental changes on aquaculture production in tropical Lake Volta, Ghana. Aquaculture International. 2018;26(6):1387-400. https://doi.org/10.1007/s10499-018-0294-7

62. Nunoo FKE, Asamoah EK, Osei-Asare YB. Economics of aquaculture production: a case study of pond and pen culture in southern Ghana. Aquaculture Research. 2014;45(4):675-88. https://doi.org/10.1111/are.12003

63. Ofori JK, Abban EK, Karikari AY, Brummett RE. Production Parameters and Economics of Small-Scale Tilapia Cage Aquaculture in the Volta Lake, Ghana. Journal of Applied Aquaculture. 2010;22(4):337-51. https://doi.org/10.1080/10454438.2010.527591

64. Consulting L. Market Study of the Aquaculture Market in Kenya - Kenya Market-led Aquaculture Programme (KMAP). 2016:1-75. Available from: https://www.farmafrica.org/downloads/study-of-the-kenyan-aquaculture-market.pdf

65. KMFRI. Kenya Aquaculture Brief 2017: Status, Trends, Challenges and Future Outlook. Kenya Marine and Fisheries Research Institute. 2017. Available from: https://www.kmfri.co.ke/images/pdf/Kenya_Aquaculture_Brief_2017.pdf

66. Kundu R, Muchiri M, Njiru M, Nyamweya C. Effect of Social and Economic Drivers on Success of Small Scale Fish farming in Western Kenya. African Journal of Tropical Hydrobiology and Fisheries. 2016(14):29-44.

67. Munguti JM, Kim J-D, Ogello EO. An Overview of Kenyan Aquaculture: Current Status, Challenges, and Opportunities for Future Development. Fisheries and aquatic sciences. 2014;17(1):1-11. https://doi.org/10.5657/fas.2014.0001

68. Ndanga LZB, Quagrainie KK, Dennis JH. Economically feasible options for increased women participation in Kenyan aquaculture value chain. Aquaculture. 2013;414-415:183-90. https://doi.org/10.1016/j.aquaculture.2013.08.012

69. Neira I, Engle CR, Ngugi C. Economic and Risk Analysis of Tilapia Production in Kenya. Journal of Applied Aquaculture. 2009;21(2):73-95. https://doi.org/10.1080/10454430902892842

70. Opiyo MA, Marijani E, Muendo P, Odede R, Leschen W, Charo-Karisa H. A review of aquaculture production and health management practices of farmed fish in Kenya. Int J Vet Sci Med. 2018;6(2):141-8. https://doi.org/10.1016/j.ijvsm.2018.07.001

71. Shikoli WP, Mukoya-Wangia S, Joyce G M, Jesse T N, Nicholas S. Enabling environment for fish farming as an alternative livelihood in Makueni, Kenya. International

Journal of Fisheries and Aquatic Studies. 2017;5(2):679-83. Available from: https://www.fisheriesjournal.com/archives/2017/vol5issue2/PartI/5-1-11-257.pdf

72. Wambua MM. A cost–benefit analysis of the fish farming enterprise productivity program project in Kenya. The case of implementation of the aquaculture development component in Meru County. United Nations University Fisheries Training Programme, Iceland [final project]. 2018. Available from: http://www.unuftp.is/static/fellows/document/moses15prf.pdf

73. Limuwa M, Singini W, Storebakken T. Is Fish Farming an Illusion for Lake Malawi Riparian Communities under Environmental Changes? Sustainability. 2018;10(5). https://doi.org/10.3390/su10051453

74. Mussa H, Kaunda E, Banda L. Economic analysis of small-scale fish farming in Bunda, Lilongwe, Malawi. RUFORUM Working Document Series. 2016;14(2):855 - 60. Availabe from: https://www.cabdirect.org/cabdirect/abstract/20193088337

75. Phiri F, Yuan X. Economic Profitability of Tilapia Production in Malawi and China. Journal of Aquaculture Research & Development. 2018;09(05). https://doi.org/10.4172/2155-9546.1000535

76. Oluwatobi AA, Mutalib HA, Adeniyi TK, Olabode JO, Adeyemi A. Possible Aquaculture Development in Nigeria: Evidence for Commercial Prospects. Journal of Agricultural Science and Technology B. 2017;7(3):194-205. https://doi.org/10.17265/2161-6264/2017.03.007

77. Ayinla OA. Analysis of feeds and fertilizers for sustainable aquaculture development in Nigeria. In: Hasan MR, Hecht T, Silva SSD, Tacon AGJ, editors. Study and analysis of feeds and fertilizers for sustainable aquaculture development FAO Fisheries Technical Paper No 497. Rome: FAO; 2007. p. 453-70.

78. Dauda AB, Natrah I, Karim M, Kamarudin MS, Bichi AuH. African Catfish Aquaculture in Malaysia and Nigeria: Status, Trends and Prospects. Fisheries and Aquaculture Journal. 2018;09(01). https://doi.org/10.4172/2150-3508.1000237

79. Emmanuel O, Chinenye A, Oluwatobi A, Peter K. Review of Aquaculture Production and Management in Nigeria. American Journal of Experimental Agriculture. 2014;4(10):1137-51. Available from: http://eprints.covenantuniversity.edu.ng/3561/1/AJEA%20final%20publication.pdf

80. Igoni-Egweke QN. Analysis of Value Addition in Commercial Catfish (Clarias gariepinus Heterobranchus spp.) Production in Rivers State, Nigeria. Federal University of Technology Owerri. 2018. Available from: https://africantheses.org/abstracts/4739-197615

81. FAO. National Aquaculture Sector Overview - United Republic of Tanzania. FAO Fisheries & Aquaculture. 2016. Available from: http://www.fao.org/fishery/countrysector/naso_tanzania/en

82. Ibengwe L, Sobo F. The Value of Tanzania Fisheries and Aquaculture: Assessment of the Contribution of the Sector to Gross Domestic Product. In: Taylor WW, Bartley DM, Goddard CI, Leonard NJ, Welcomme R, editors. Freshwater, fish and the future: proceedings of the global cross-sectoral conference. Rome; East Lansing; Bethesda, Maryland: Food and Agriculture Organization of the United Nations; Michigan State University; American Fisheries Society; 2011.

83. Kleih U, Kishe M, Yunus KB. Financial Services for SME Fisheries Producers in Tanzania. 2010. Available from: http://africanfisheriesinvestment.org/files/casestudies/casestudy-tanzania.pdf

84. Ministry of Agriculture Livestock and Fisheries. The Tanzanian Fisheries Sector - Challenges and opportunities. United Republic of Tanzania. 2016. Available from:

https://www.wiomsa.org/download/the-tanzanian-fisheries-sectors-challenges-and-opportunities/

85. Rukanda JJ. Evaluation of aquaculture development in Tanzania. United Nations University Fisheries Training Programme, Iceland. 2018. Available from: https://www.grocentre.is/static/gro/publication/353/document/janeth16aprf.pdf

86. Stutzman E, Molnar J, Atukunda G, Walakira J. Understanding the Role of Fish Farmer Associations as Intermediaries for the Commercialization of Aquaculture in Uganda. Fisheries and Aquaculture Journal. 2017;08(03). https://doi.org/10.4172/2150-3508.1000214

87. Genschick S, Kaminski A, Cole S, Tran N, Chimatiro S, Lundeba M. Toward more inclusive and sustainable development of Zambian aquaculture. Penang, Malaysia: CGIAR Research Program on Fish Agri-Food Systems Program Brief: FISH-2017-07. 2017. Available from: https://hdl.handle.net/20.500.12348/16

88. Musuka CG, Musonda FF. Current and future prospects of commercial fish farming in Zambia. Aquaculture, Aquarium, Conservation & Legislation International Journal of the Bioflux Society. 2012;5(1):79-87. Available from: http://bioflux.com.ro/docs/AACL_5.2.5.pdf

89. Namonje-Kapembwa T, Samboko P. Assessing the Profitability of Small-Scale Aquaculture Fish Production in Zambia. Working Paper 123 Indaba Agricultural Policy Research Institute (IAPRI). 2017. Available from: https://pdf.usaid.gov/pdf_docs/PA00SZ57.pdf

90. McDermott J, Wyatt AJ. The role of pulses in sustainable and healthy food systems. Ann N Y Acad Sci. 2017;1392(1):30-42. https://doi.org/10.1111/nyas.13319

91. Thilsted SH, Thorne-Lyman A, Webb P, Bogard JR, Subasinghe R, Phillips MJ, et al. Sustaining healthy diets: The role of capture fisheries and aquaculture for improving nutrition

in the post-2015 era. Food Policy. 2016;61:126-31. https://doi.org/10.1016/j.foodpol.2016.02.005

92. Byrd KA, Pincus L, Pasqualino MM, Muzofa F, Cole SM. Dried small fish provide nutrient densities important for the first 1000 days. Matern Child Nutr. 2021:e13192. https://doi.org/10.1111/mcn.13192

93. Hallström E, Bergman K, Mifflin K, Parker R, Tyedmers P, Troell M, et al. Combined climate and nutritional performance of seafoods. Journal of Cleaner Production. 2019;230:402-11. https://doi/org/10.1016/j.jclepro.2019.04.229

94. Troell M, Jonell M, Crona B. Scoping report: The role of seafood for sustainable and healthy diets. The EAT-Lancet Commission report through a blue lens. 2019;266:1-24. Available from: Available from: https://eatforum.org/content/uploads/2019/11/Seafood_Scoping_Report_EAT-Lancet.pdf

95. Bennett A, Basurto X, Virdin J, Lin X, Betances SJ, Smith MD, et al. Recognize fish as food in policy discourse and development funding. Ambio. 2021;50(5):981-9. https://doi.org/1007/s13280-020-01451-4

96. Jennings S, Stentiford GD, Leocadio AM, Jeffery KR, Metcalfe JD, Katsiadaki I, et al. Aquatic food security: insights into challenges and solutions from an analysis of interactions between fisheries, aquaculture, food safety, human health, fish and human welfare, economy and environment. Fish and Fisheries. 2016. https://doi.org/10.1111/faf.12152

97. Arulingam I, Nigussie L, Sellamuttu SS, Debevec L. Youth participation in small-scale fisheries, aquaculture and value chains in Africa and the Asia-Pacific. Penang, Malaysia: CGIAR Research Program on Fish Agri-Food Systems. Program Report: FISH-2019-14. 2019. Available from: https://hdl.handle.net/20.500.12348/3937

98. African Development Bank. Jobs for youth in Africa strategy: Regional ministerial conferences on youth employment and entrepreneurship in Africa. Abidjan, Côte d'Ivoire: African Development Bank Group. 2017. Available from: https://www.afdb.org/fileadmin/uploads/afdb/Documents/Generic-Documents/Ministerial_Conferences_Report_En.pdf

99. Filmer D, Fox L. Youth Employment in Sub-Saharan Africa. Africa Development Forum, World Bank Washington, DC. 2014. https://doi.org/10.1596/978-1-4648-0107-5

100. FAO. The State of World Fisheries and Aquaculture 2020. Sustainability in action. Rome. 2020. Available from: https://doi.org/10.4060/ca9229en

101. Moberg E, Allison EH, Harl HK, Arbow T, Almaraz M, Dixon J, et al. Combined innovations in public policy, the private sector and culture can drive sustainability transitions in food systems. Nature Food. 2021. https://doi.org/10.1038/s43016-021-00261-5

102. Kleiber D, Harris LM, Vincent ACJ. Gender and small-scale fisheries: A case for counting women and beyond. Fish and Fisheries. 2015;16(4):547-62. https://doi.org/10.1111/faf.12075

Figure 1

Click here to access/download;Figure;Figure 1.tif

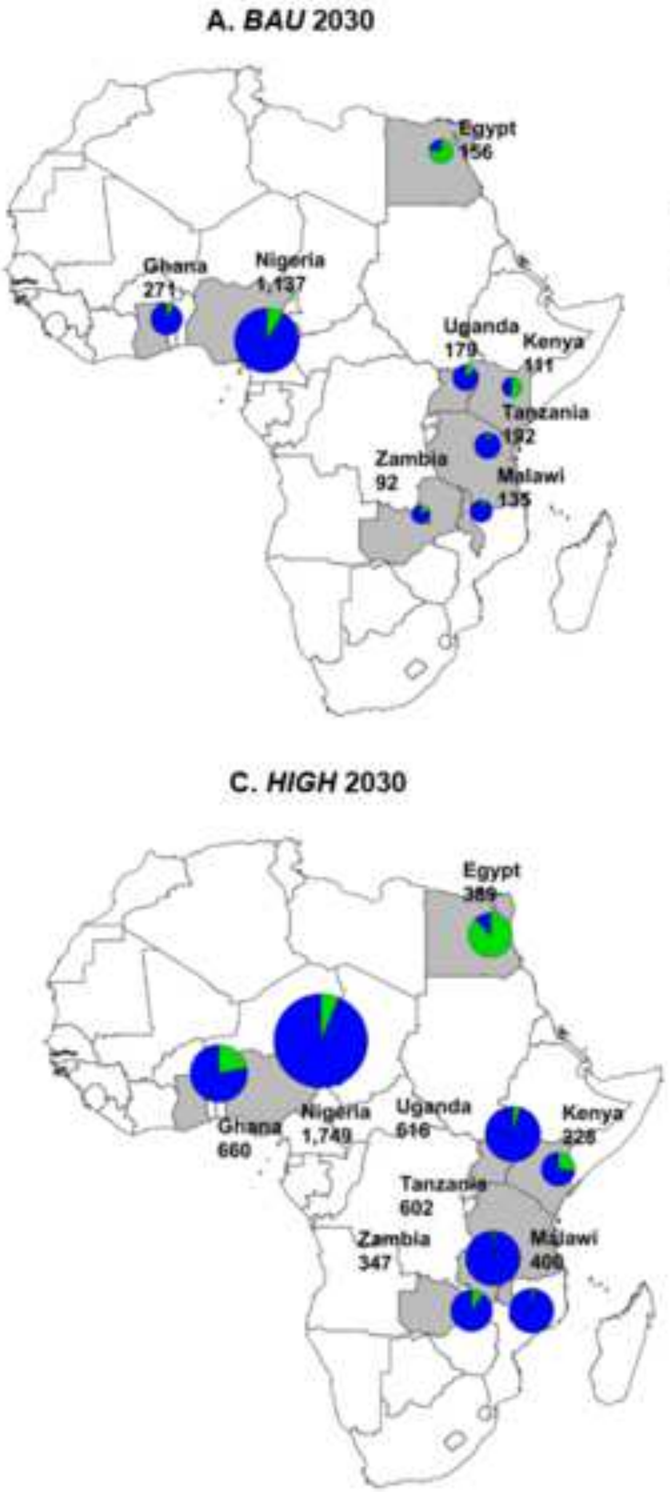

Figure 2 Click here to access/download;Figure;Figure 2.tif

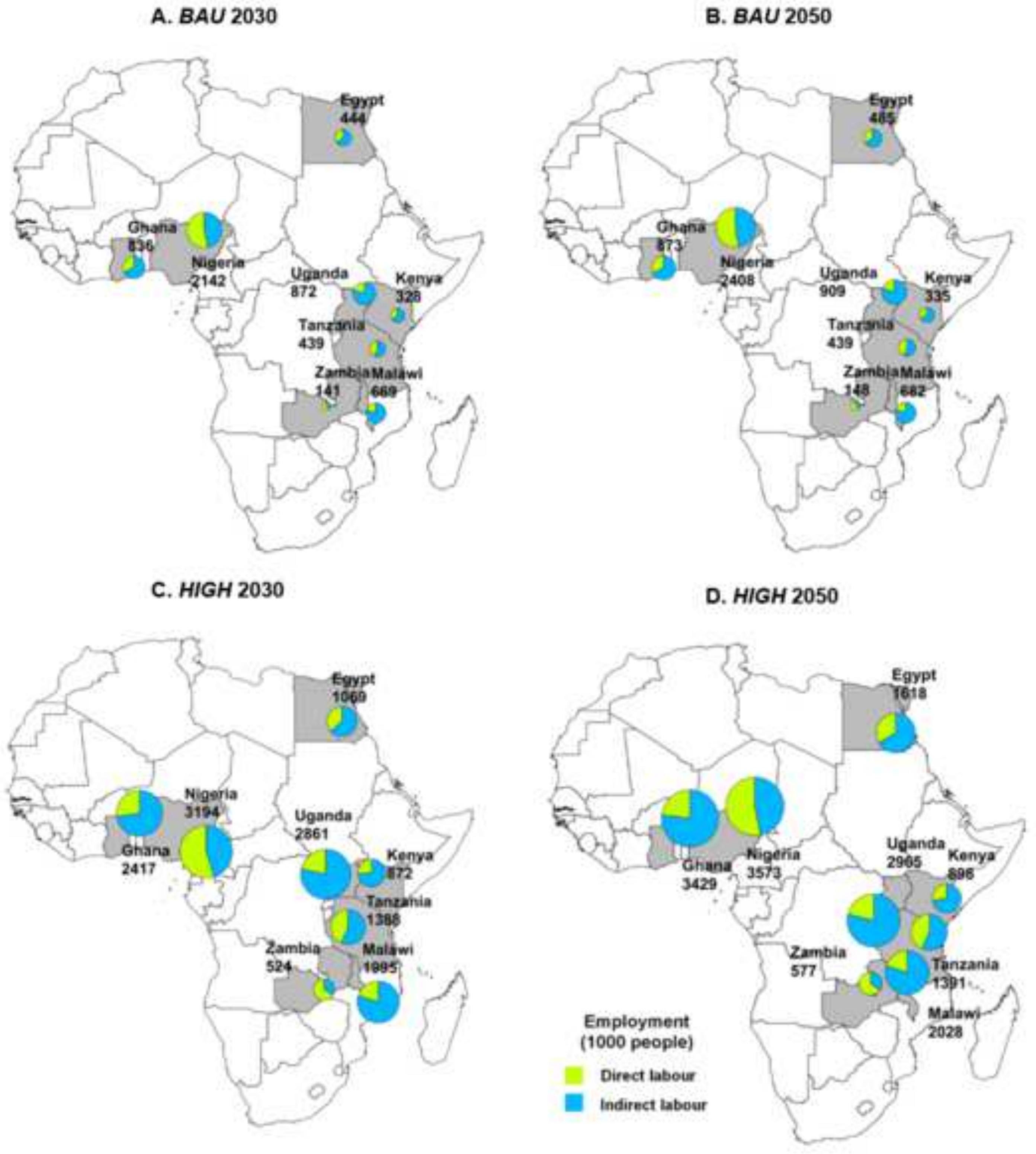

Click here to access/download;Figure;Figure 3.tif

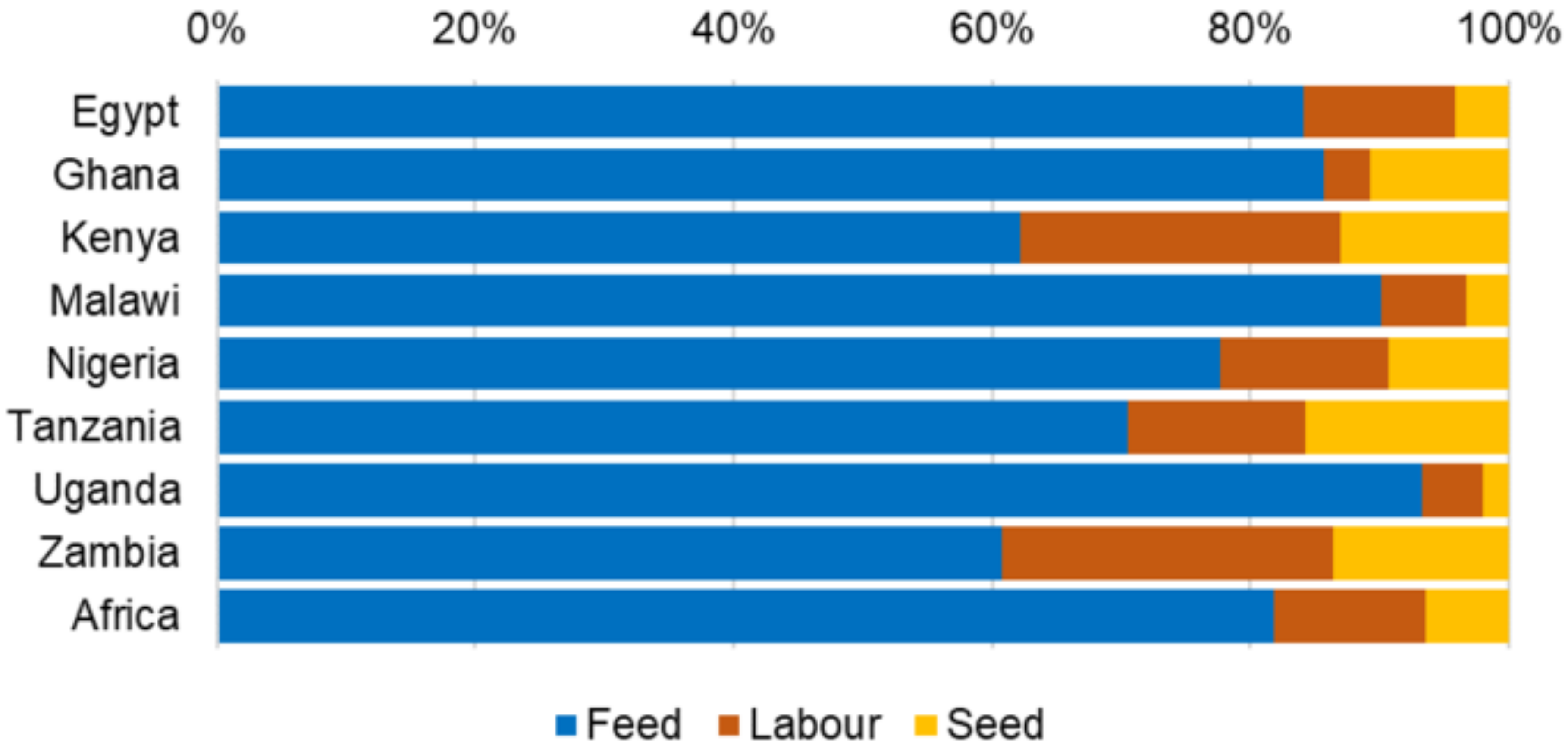

Supplement: Attachment — Submitted filename: PONE-D-21-16152_reviewer-KO.pdf [file pone.0261615.s001.pdf]
