# Supplementary material for: The future of fish in Africa: Employment and investment opportunities
Source: PLoS One. 2021 Dec 22;16(12):e0261615. doi: 10.1371/journal.pone.0261615 (PMC8694441; doi:10.1371/journal.pone.0261615)
Supplement: Attachment — Submitted filename: future of fish for Africa reviewed aticle submitted to plusone review.pdf [file pone.0261615.s002.pdf]

The article is of importance to Africa's future fish production. However, the following corrections/clarifications are needed

The objectives of the study is not clear under Abstract section.

Line 27 had different font characteristic

Line 30-31 not clear which one get 26 people, what system? And what specific contribution from each of feed, seed and labour

Line 51: I Africa?

Line 55-59: has too long sentence and there are such all over the manuscript. These has to be corrected

The use of comma, tenses and grammar as observed for instance in lines 65, 93, 117, 178, 202 (growth?), 246, 266, 336

There is need for ref in citing in some cases such as line 107,

Lines 100-107also 110-112 sounds line methods being presented in introduction

It would be good to highlight the % of each country, the criteria for setting the ratio and how the experts look like (criteria for their selection /area of experts - aquaculture/fisheries?

Table 1 seems not clear. Justify why population growth ranged 5years, GDP growth ranged10years, undernourishment only captured 2018, unemployment 2018. These variation need to be backed up with good reasons

Data being referred in Line 137-139 need to be cited

Line141: what is the citation for the "Shared Socio Economic Pathways 2"

Line 142- need to explain the assuming "slow pace environmental degradation. Explain what informed moderate challenges from climate change adaptation.

Line 147 seems to discuss aquaculture and fisheries together. Does it mean that data on the two were not separated. Check FAO data for separate figure for each of them.

Line 153-154. You need to support the choice of the listed species with good reason. You would need to provide information on the % Africas aquaculture being contributed by mullet and pangasius? What brought their importance for projection in Africa. There are similar issues in line 163-164, 193 (they all need justification).

Line 200 rather use "broodstock" instead of "fertilized eggs"

Table 2; each column has cited many references but singler value was presented for each column, no variation/ standard deviation? In am curious why current values were not mentioned at all even in the discussion? Does it mean that the dynamics of these variants were held constant till now?

The study has too many limitations listed. It could be referred "prelimnary"
