# Supplementary material for: The future of fish in Africa: Employment and investment opportunities
Source: PLoS One. 2021 Dec 22;16(12):e0261615. doi: 10.1371/journal.pone.0261615 (PMC8694441; doi:10.1371/journal.pone.0261615)
Supplement: Attachment — Submitted filename: Response to Reviewers.docx [file pone.0261615.s004.docx]

**RESPONSE TO REVIEWERS**

We thank the reviewers’ comments and helpful advice. We responded to the minor comment, and made changes in the revised version (revised manuscript with track changes).

**Reviewer #3 Comments**

**Q1**: The reviewer comments were adequately addressed. One small suggestion for an edit: the final paragraph contains the following confusing newly added sentence:
"The current IMPACT model adopts highly aggregated with sixteen fish categories on the supply side and nine fish categories on the demand side." Perhaps "Given the high diversity in fished and cultured species in Africa, the current IMPACT model is highly aggregated with sixteen fish categories on the supply side and nine fish categories on the demand side." At least, if this was what was meant.

**A1**: Thank you for your comments. We have revised the sentence as follow:

Given the high diversity in wild-caught and cultured fish species in Africa and worldwide, the current IMPACT model is highly aggregated with sixteen fish categories on the supply side and nine categories on the demand side.
